# Natural variation and genetic make-up of leaf blade area in spring barley

**DOI:** 10.1007/s00122-018-3053-2

**Published:** 2018-01-19

**Authors:** Ahmad M. Alqudah, Helmy M. Youssef, Andreas Graner, Thorsten Schnurbusch

**Affiliations:** 1 HEISENBERG-Research Group Plant Architecture, Leibniz Institute of Plant Genetics and Crop Plant Research (IPK), Corrensstrasse 3, OT Gatersleben, 06466 Seeland, Germany; 2 Faculty of Agriculture, Cairo University, Giza, 12613 Egypt; 3 Research Group Genome Diversity, Leibniz Institute of Plant Genetics and Crop Plant Research (IPK), Corrensstrasse 3, OT Gatersleben, 06466 Seeland, Germany

## Abstract

**Key message:**

GWAS analysis for leaf blade area (LA) revealed intriguing genomic regions associated with putatively novel QTL and known plant stature-related phytohormone and sugar-related genes.

**Abstract:**

Despite long-standing studies in the morpho-physiological characters of leaf blade area (LA) in cereal crops, advanced genetic studies to explore its natural variation are lacking. The importance of modifying LA in improving cereal grain yield and the genes controlling leaf traits have been well studied in rice but not in temperate cereals. To better understand the natural genetic variation of LA at four developmental stages, main culm LA was measured from 215 worldwide spring barleys including 92 photoperiod-sensitive accessions [*PHOTOPERIOD RESPONSE LOCUS 1* (*Ppd*-*H1*)] and 123 accessions with reduced photoperiod sensitivity (*ppd*-*H1*) locus under controlled greenhouse conditions (long-day; 16/8 h; ~ 20/~ 16 °C day/night). The LA of *Ppd*-*H1*-carrying accessions was always smaller than in *ppd*-*H1*-carrying accessions. We found that nine SNPs from the *Ppd*-*H1* gene were present in the collection of which marker 9 (M9; G/T in the CCT-domain) showed the most significant and consistent effect on LA at all studied developmental stages. Genome-wide association scans (GWAS) showed that the accessions carrying the *ppd*-*H1* allele T/M9 (late heading) possessed more genetic variation in LA than the *Ppd*-*H1* group carrying G/M9 (early heading). Several QTL with major effects on LA variation were found close to plant stature-related heading time, phytohormone- and sugar-related genes. The results provide evidence that natural variation of LA is an important source for improving grain yield, adaptation and canopy architecture of temperate cereals.

**Electronic supplementary material:**

The online version of this article (10.1007/s00122-018-3053-2) contains supplementary material, which is available to authorized users.

## Introduction

Leaf blade area (LA) is considered as one of the major drivers of plant architecture that in turn influences the adaptations to environmental cues and grain yield. Donald (1968) and Jennings (1964) proposed a model for enhancing grain yield potential by modifying traits such as LA in cereals using an “ideotype” breeding. LA has a direct impact on crop stature, growth and yield through photosynthesis (Chen et al. 1995) which can be maximized by expanding photosynthetic-LA in rice, sorghum and wheat (Driever et al. 2014; Jiang et al. 2015; Kebrom and Mullet 2015). Factually, the leaf is the major photosynthetic organ that supplies assimilates for regulating plant stature, e.g. photosynthetic-LA is tightly linked with sugar production that in turn regulates shoot branching in sorghum (Evers 2015; Kebrom and Mullet 2015). The position of individual leaves affects their contribution to grain yield, especially the flag leaf, which is considered as the main source of carbohydrate synthesis in barley (Tao 1999). The relationship between flag-LA and yield traits was intensively studied in rice (Wang et al. 2011) and barley (Tao 1999). Moreover, the genetic analysis of flag-LA during grain filling had been studied in rice (Zhang et al. 2015) and barley (Yang and Lu 1991). Alqudah and Schnurbusch (2015) identified a high contribution of main culm LA on single plant yield in barley and showed the quantitative inheritance of LA at pre-anthesis developmental stages under different environments. Greenhouse (GH) conditions and single plant analysis were found to be suitable for studying leaf developmental traits because they maximized phenotypic/genetic variation among accessions (Alqudah and Schnurbusch 2015). Therefore, to study LA progression during pre-anthesis development is important to better understand the genetic link between LA and phase duration.

The embryo of the barley grain has up to four leaf primordia covering the young shoot apical meristem (SAM), whereas further leaf primordia can be produced after germination under non-inductive conditions (Kirby and Appleyard 1987). The newly induced leaf primordia arise as a result of periclinal cell division in the dermatogen cells of the SAM, which is followed by periclinal and anticlinal cell divisions and expansions in the dermatogen and the hypodermal cells at the leaf primordia (Sharman 1945). Subsequently, the leaf primordium converts into a mature leaf through promoting cell division and expansion (Gonzalez et al. 2012). The above-mentioned phases are overlapping and interconnected with impact on final leaf size, which is strictly controlled through spatial–temporal genetic factors (Gonzalez et al. 2012). In barley, the duration of leaf initiation and developmental phases are variable across genotypes and genetically inherited (Kernich et al. 1995; Kirby and Riggs 1978). Gonzalez et al. (2012) reported that the duration of leaf initiation and developmental phases, especially the cell expansion phase, affect final LA. The duration of leaf elongation is considered to be one of the major determinants of grass LA (Chenu et al. 2008; Voorend et al. 2014) that in turn explains the variation in response to environmental cues or across genotypes (Tardieu et al. 2000). Measuring LA during aforementioned developmental times was proposed to be an adequate tool to describe the analysis of kinematic parameters such as cell division, expansion, size and density (Gonzalez et al. 2012).

Quantitative trait loci (QTL) analysis and identification of genes underlying leaf size and shape traits are well advanced in rice. Most of the QTL studies in barley and rice were focused on flag-LA and related traits (Bing et al. 2006; Gyenis et al. 2007; Xue et al. 2008; Zhang et al. 2015). In addition to QTL analyses, several genes controlling flag leaf traits have been identified in rice, such as *SEMI*-*ROLLED LEAF 1* (*SRL1*), Xiang et al. (2012); *SHALLOT*-*LIKE 1* (*SLL1*), Zhang et al. (2009); *NARROW LEAF 1* (*NAL1*), Qi et al. (2008); *NARROW AND ROLLED LEAF 1* (*NRL1*), Hu et al. (2010) and *NARROW LEAF 7* (*NAL7*), Fujino et al. (2008). By mutant analysis of these genes, it was found that abnormal leaf shape and size resulted from abnormal cell division and/or expansion. For instance, Jiang et al. (2015) found that leaf length and width were reduced by 50% in *nal1*-*2* and *nal1*-*3* rice mutants as a result of cell division suppression. Aberrant periclinal cell divisions lead to increased cell layers in the leaf epidermis of barley *elongation* (*elo2* and *elo5*) mutants (Lewis et al. 2009) and *Extra cell layers 1* (*Xcl1*) maize mutant (Kessler et al. 2002). Recently, Jöst et al. (2016) cloned the barley *BROAD LEAF 1* (*BLF1*) gene encoding an INDETERMINATE DOMAIN protein that limits cell divisions as a negative regulator of leaf-width during leaf primordia outgrowth. Barley *NARROW LEAFED DWARF1* that encodes a WUSCHEL-RELATED HOMEOBOX 3 (WOX3) regulates the development of the marginal regions in the leaves had been recently cloned by Yoshikawa et al. (2016).

A genome-wide association study (GWAS) was implemented in a nested association mapping in maize to understand the second top leaf (penultimate leaf) architecture at flowering stage (Tian et al. 2011). Allelic variation of the *PHOTOPERIOD RESPONSE LOCUS 1* (*Ppd*-*H1*) gene was associated with natural variation in leaf size in European winter barley cultivars (Digel et al. 2016). Most of the previous genetic studies focused on the flag leaf or single leaf at a specific stage; but heretofore no genetic study considered the natural variation of LA at different developmental stages using GWAS analysis. Performing such study may provide general implications related to the prevalence of natural genetic variation for LA in cereals.

*PHOTOPERIOD RESPONSE LOCUS 1* (*Ppd*-*H1*) is one of the central genes controlling heading time in barley where amino acid changes at CCT [CONSTANS, CO-LIKE, TIMING OF CAB1 (TOC1)]-domain separated *Ppd*-*H1* into photoperiod-sensitive (G allele) and *ppd*-*H1* reduced photoperiod sensitivity [T allele; Turner et al. (2005)]. These alleles were used to account for population structure to study the genetic variation of phase duration and plant stature in a worldwide spring barley collection (Alqudah et al. 2014, 2016). Following this approach, main culm LA-blades were collected from the studied accessions at four developmental stages at awn primordium (AP), tipping (TIP), heading (HD) and anther extrusion (AE) stages. The 215 tested accessions were grown under controlled greenhouse (GH) conditions. For GWAS analysis, the 9K gene-based single nucleotide polymorphism (SNP) chip provided a high-resolution genetic map. Here we present the first GWAS study of LA in temperate cereals, showing that a single variant at the *Ppd*-*H1* locus marker 9 (M9) sitting in the CCT-domain had the most significant and consistent effect on LA variation at all studied developmental stages, consistent with Digel et al. (2016). Further GWAS analyses from the two photoperiod groups (i.e. *Ppd*-*H1*/*ppd*-*H1*) disclosed associations with enriched genomic regions co-locating with putative candidate genes controlling leaf traits known in other crop species and/or novel QTL. Therefore, studying LA is important to explore the influence of allelic variation on the natural genetic variation of LA in barley.

## Materials and methods

### Plant material and population structure

**Fig. 1:**
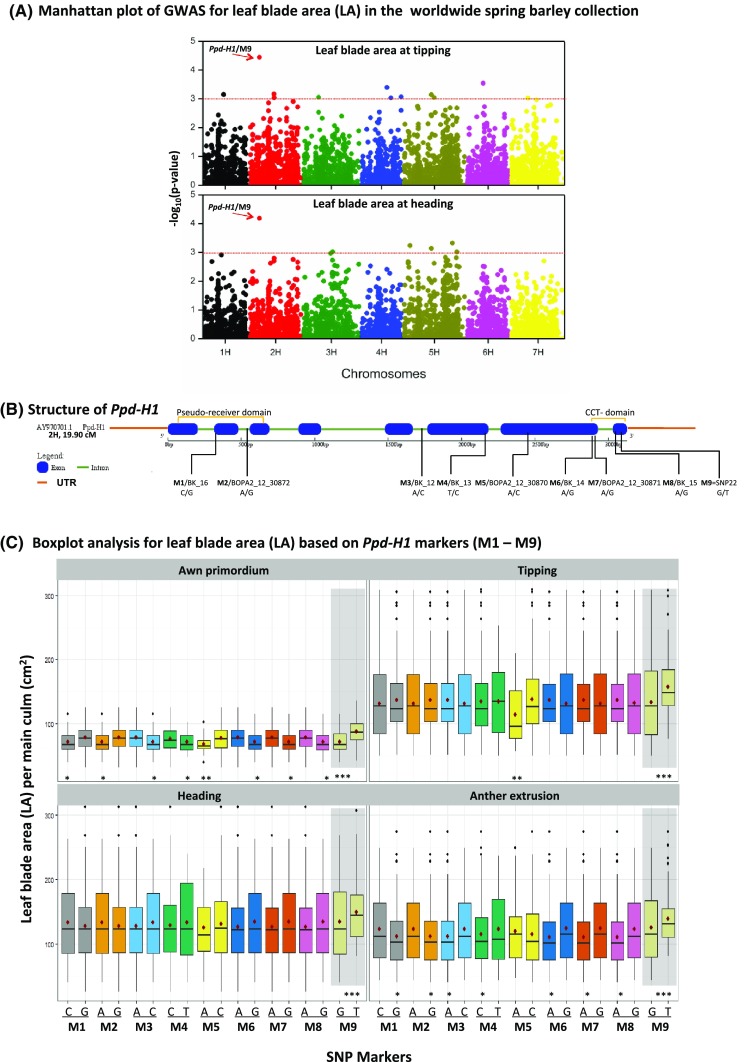

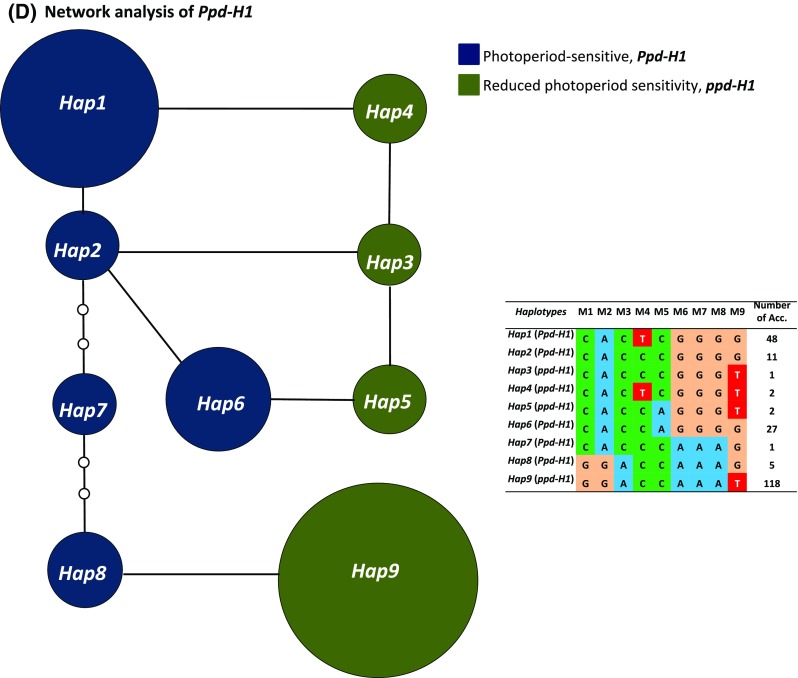
**a** Manhattan plot of GWAS for leaf blade area at tipping and heading stages; the red-arrow indicates the location of M9 at chromosome 2HS, and the red dotted line denote the threshold significance level, − log_10_ (*P* value of  0.001). **b** *Ppd*-*H1* gene structure. **c** Box plot analysis of leaf area based on the nine SNPs derived from *Ppd*-*H1*. The degree of significance indicated as *P, 0.05; **P, 0.01; ***P, 0.001. Significant differences (*P* ≤ 0.05) were determined using LSD. Significant differences between the alleles of each marker were calculated at each developmental stage separately. **d** Network analysis of *Ppd*-*H1* in 215 worldwide spring barley accessions, dark-blue for photoperiod-sensitive haplotypes and dark-green for reduced photoperiod sensitivity. Three biological replicates were used from each accession at each pre-anthesis developmental stage (*n* = 92 and 123 for photoperiod sensitive and reduced photoperiod sensitivity barley, respectively)

A diverse spring barley collection of 215 worldwide accessions was used in this study. The accessions had previously been genotyped using high-density 9K SNPs chip from Illumina (Alqudah et al. 2014, 2016). Platform assayed 6355 SNPs that includes eight markers localized in *Ppd*-*H1* gene (BOPA2_12_30870, BOPA2_12_30871, BOPA2_12_30872, BK_12, BK_13, BK_14, BK_15, BK_16) in addition to M9/SNP22 (G/T, Turner et al. (2005), Fig. 1a). *Ppd*-*H1* has been positionally cloned and includes eight exons with pseudo-receiver domain and CCT domain, Turner et al. (2005). *Ppd*-*H1* is the only barley highly confidence gene MLOC_81154.10 (HORVU2Hr1G013400) located in morex_contig_94710 with all above mentioned iSELECT markers at 2H (19.9 cM) based on the recently published high-quality reference genome assembly (Mascher et al. 2017). Population stratification of the germplasm panel was previously published and structured based on M9/SNP22 (G/T) (Alqudah et al. 2014, 2016). Network analysis of nine *Ppd*-*H1* SNP haplotypes was carried out using TCS v1.21 software (http://darwin.uvigo.es/software/tcs.html; Clement (2000) in 215 spring barley accessions (Fig. 1b). To find more information about the accessions’ origins and photoperiod status are present in Table S1.

### Phenotype data

Thirty plants from each of the 215 spring barley accessions were grown under GH conditions (long-day: LD condition, 16/8 h day/night and ~ 20/~ 16 °C day/night) at the Leibniz Institute of Plant Genetics and Crop Plant Research (IPK) in Gatersleben, Germany (51°49′23″N, 11°17′13″E, altitude 112 m). Details about how the accessions of the population were grown have been previously described (Alqudah et al. 2014). LA of completely unfolded and fully developed leaf blades from the main culm was harvested by hand (all leaves together) at different developmental stages. (AP: awn primordium, Alqudah and Schnurbusch (2014); TIP: tipping, Z49; HD: heading, Z55; AE: anther extrusion, Z65; Hrv: Harvesting, Zadoks et al. (1974). Then the total leaf blade area of the main culm (LA, cm^2^) was measured by LA-meter (portable Li-COR area meter, Li-3000) as described by Alqudah and Schnurbusch (2015). The data were collected from three randomly selected biological replicates per accession. Summary statistics for LA in each group at different developmental stages were determined by GenStat 16 (GenStat 2014). Analysis of variance (ANOVA) was conducted to compare the differences between accessions, geographic region and groups (*Ppd*-*H1* vs. *ppd*-*H1*) using GenStat 16 (GenStat 2014) at probability level *P* ≤ 0.05. Means were separated according to the Fisher’s least significant difference (LSD) at *P* ≤ 0.05 levels of probability. Best linear unbiased estimates (BLUEs) was used to estimate each accession’s phenotypic means, which in turn were used in the association analysis while residual maximum likelihood (REML) was used to analyze phenotypic data (GenStat 2014). Broad-sense heritability for LA in each group was calculated across growth times as the ratio between the genetic variance and the phenotypic variance components using GenStat 16 (GenStat 2014).

### Association analysis

GWAS of each *Ppd*-*H1* group was performed for LA at each developmental stage using the markers those passing marker-mining tests. Associations between estimated phenotypic traits (BLUEs) and each single marker was calculated using mixed linear model (MLM) in GenStat 16 (GenStat 2014). Eigen-analysis was used as a correction of population structure in each sub-population (Price et al. 2006). The strategy of GWAS analysis, allele mining, allele effect, validation of association [false discovery rate (FDR 0.001)] and creating QTL-association map have been described by Alqudah et al. (2014). Average linkage disequilibrium (LD) for the germplasm panel was previously determined (± 5 cM; Pasam et al. 2012) and used as a confidence interval to determine associated QTL. The Barke × Morex RIL POPSEQ population was used to find the genetic position anchored by physical map positions of highly associated SNP markers (SNPs ≥ FDR) and putative orthologous barley genes (characterized for LA phenotypes in other species) (Mascher et al. 2013). The sequences of highly associated markers (which show a consistent effect on LA, SNPs ≥ FDR) were blasted on the barley genome dataset and gene set (BARLEX; http://apex.ipk-gatersleben.de). The most significant hit was selected to obtain the corresponding genes, transcripts and gene annotation. Functional annotations of the candidate genes were also confirmed using known function in other cereal crops such as rice. Detailed information about these genes, their Genbank accession numbers, and genetic chromosome positions are available in Table S2.

## Results

### GWAS reveals a specific *Ppd*-*H1* haplotype as major driver for LA variation

GWAS analyses showed several genomic regions associated with LA at TIP and HD stages (Fig. 1a) with the most significant and consistent association signal at *Ppd*-*H1*/M9 (Fig. 1a). Based on the physical position of the SNP markers, nine markers (M1–M9) are physically localized within the *Ppd*-*H1* gene (Fig. 1b). M9 was located in the CCT domain and produced one amino acid change [Gly-to-Trp, G/T, Turner et al. (2005)] that defines the *ppd*-*H1* allele (T/M9; reduced photoperiod sensitivity allele). Phenotypic analysis for LA based on the nine *Ppd*-*H1* markers (M1–M9) showed significant differences in LA between the alleles of each marker at the studied developmental stages (Fig. 1c). The results clearly indicated that M9 had the highest effect (***P, 0.001) among the *Ppd*-*H1*-derived markers at all developmental stages (Fig. 1c). The accessions carrying the T/M9 allele (reduced photoperiod sensitivity allele) had larger LA than the accessions carrying photoperiod-sensitive allele G/M9 (***P, 0.001; Fig. 1c). To further explore sequence variation at the *Ppd*-*H1* locus, haplotype analysis of the spring barley accessions using these nine SNPs was performed (Fig. 1d). Five haplotypes (*Hap1*, *2*, *6*, *7* and *8*) were found in 92 accessions carrying sensitive alleles of *Ppd*-*H1*. The other four haplotypes (*Hap3*, *4*, *5* and *9*) were represented in 123 accessions carrying the insensitive allele of *ppd*-*H1*. Notably, the majority of these four insensitive haplotypes, i.e. Hap3, 4, 5 and 9, are mainly from EU (91 accessions) (Fig. 1d). The analyses of the nine markers derived from *Ppd*-*H1* (Fig. 1a, c, d) clearly indicated that the population can be divided into two groups for photoperiod responses based upon M9. Therefore, GWAS analyses were conducted for the two groups separately; accessions carrying the reduced photoperiod sensitivity *ppd*-*H1* allele T/M9 (123 accessions) and a group of 92 accessions carrying the photoperiod-sensitive allele *Ppd*-*H1*G/M9 allele [Figure S1 (Alqudah et al. 2014, 2016)].

**Fig. 2:**
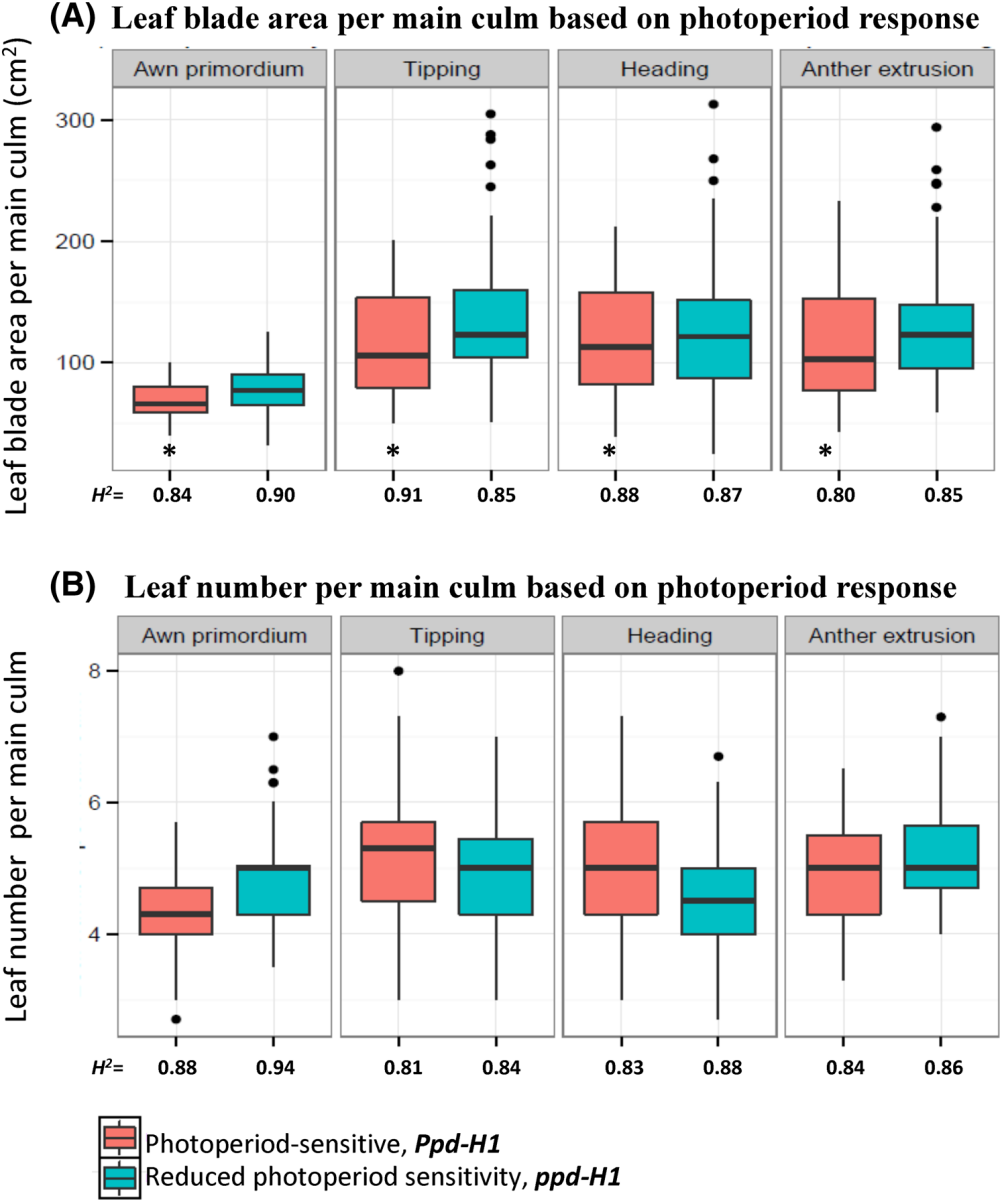
Box plot analysis of leaf blade area (**a**) and leaf number (**b**) per the main culm based on photoperiod response. *n* = 92 accessions of photoperiod sensitive (*Ppd*-*H1*) and *n* = 123 accessions with reduced photoperiod sensitivity (*ppd*-*H1*). *AP* awn primordium, Alqudah and Schnurbusch (2014); TIP: tipping, Z49; HD: heading, Z55; AE: anther extrusion, Z65; Hrv: Harvesting, Zadoks et al. (1974). Asterisk denotes leaf area significantly different at *P* ≤ 0.05 according to LSD between photoperiod groups at the same developmental stage. The degree of significance indicated as *P, 0.05; **P, 0.01; ***P, 0.001

Phenotypic analysis of 215 worldwide spring barley accessions showed that the accessions significantly varied (at *P* < 0.001) in main culm LA at all developmental stages (Table S3). Interestingly, significant differences in LA among the geographic regions of accessions were found at all developmental stages (Table S3). Photoperiod status of accessions (i.e. *Ppd*-*H1*/*ppd*-*H1*) was associated with variation in LA at all developmental stages (Table S3). These findings indicated that LA in 215 accessions was influenced by photoperiod status and/or geographical regions. Maximum LA was reached at TIP/HD; thereafter LA decreased especially in photoperiod sensitive accessions (Fig. 2a). Accessions carrying sensitive alleles at *Ppd*-*H1* showed significantly smaller LA than *ppd*-*H1* accessions (at *P* < 0.05) at all developmental stages (Fig. 2a). For leaf number, there were no significant differences between photoperiod groups (at *P* < 0.05) at all developmental stages (Fig. 2b), therefore we excluded it from further genetic analyses. The correlation analysis between LA and leaf number per the main culm across all developmental stages showed a moderate positive relationship (Figure S2).

**Fig. 3:**
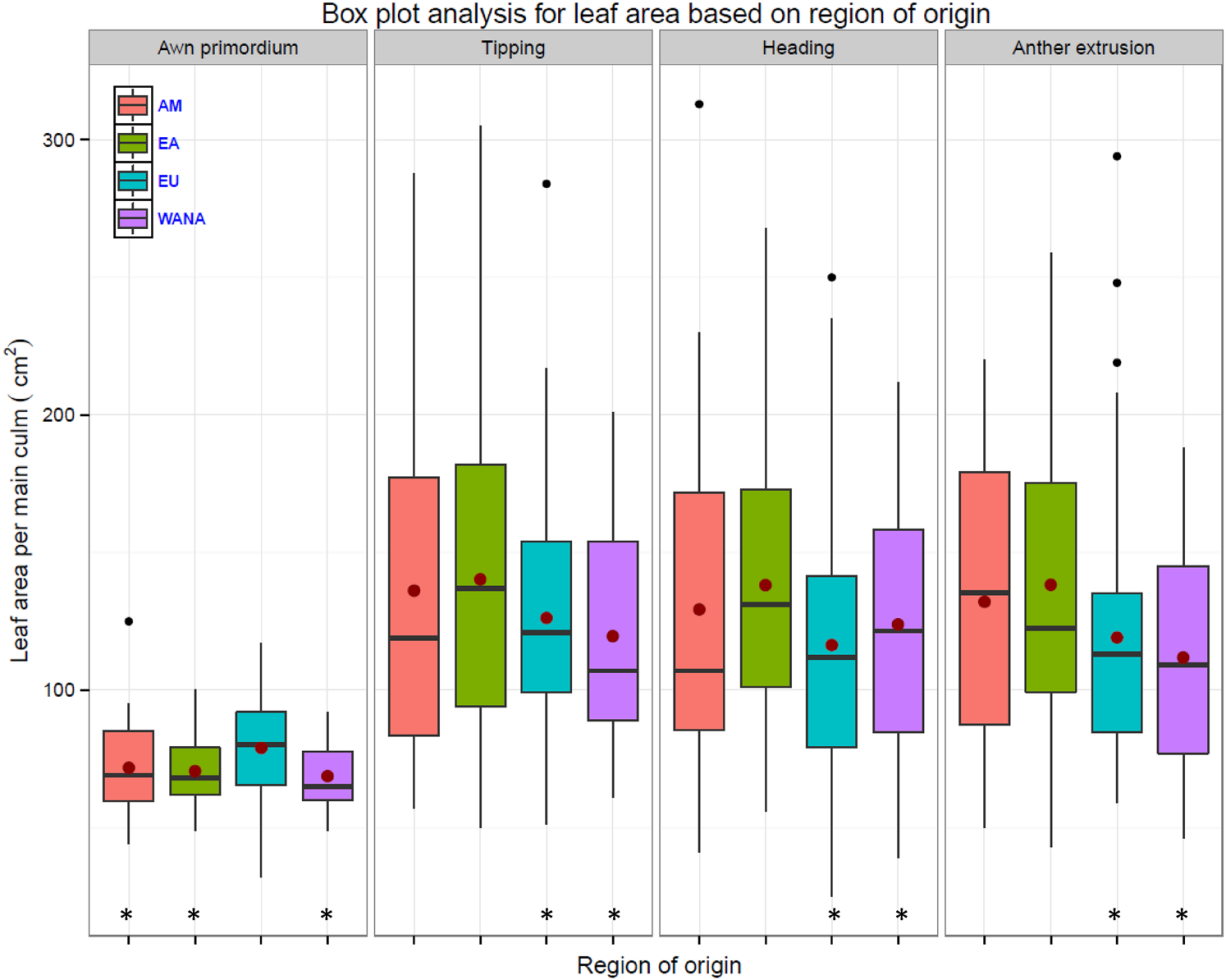
Box plot analysis of leaf blade area per the main culm at different developmental stages of 215 spring barley accessions from the different geographical region. Asterisk denotes leaf area significantly different at *P* ≤ 0.05 according to LSD between geographical region at the same developmental stage. The degree of significance indicated as *P, 0.05; **P, 0.01; ***P, 0.001 (*n* = 92 and 123 for *Ppd*-*H1* and *ppd*-*H1* barleys, respectively). Number of accessions for WANA = 45, EU = 105, EA = 37 and AM = 28

Based on the geographic region of the accessions (including the accessions from both photoperiod groups), European (EU) accessions had significantly larger LA at AP (Fig. 3) followed by accessions from East Asia (EA). There were no significant differences between the regions of the origin in LA within the *Ppd*-*H1* group at all developmental stages (Figure S3a). Whereas the accessions from EA and Americas (AM) had the largest LA in comparison with accessions from EU, West Asia and North Africa (WANA) in the *ppd*-*H1* group (Figure S3b). Leaf number per main culm was not significantly different between the geographic regions of the accessions (Figure S4).

The genetic variation in LA at the TIP stage between the accessions was explained in the principle component analysis (PCA) using 6355 SNPs. The accessions were almost separated into *Ppd*-*H1* and *ppd*-*H1* spring barleys (Figure S5) confirming the population structure of the collection (Figure S1). The broad-sense heritability values for LA at the developmental stages in each photoperiod group (*Ppd*-*H1* and *ppd*-*H1*) were above 0.75 (Fig. 2a), indicating that LA is a highly heritable trait at early- and late-developmental stages and under these growth conditions, which helped us to detect particular QTL for each stage within each photoperiod group.

### Natural genetic variation of LA and marker-trait association using GWAS at four developmental stages in two photoperiod groups

A GWAS was performed at AP, TIP, HD, and AE developmental stages for each photoperiod group (*Ppd*-*H1* and *ppd*-*H1*) independently using 9K array and mixed models approach (Figure S6). The GWAS analysis showed that most of the significant associations appeared after AP stage, suggesting that there is ample natural phenotypic/genetic variation for LA during late-reproductive phases (after AP to AE).

#### QTL for LA in the *Ppd-H1* group

**Fig. 4:**
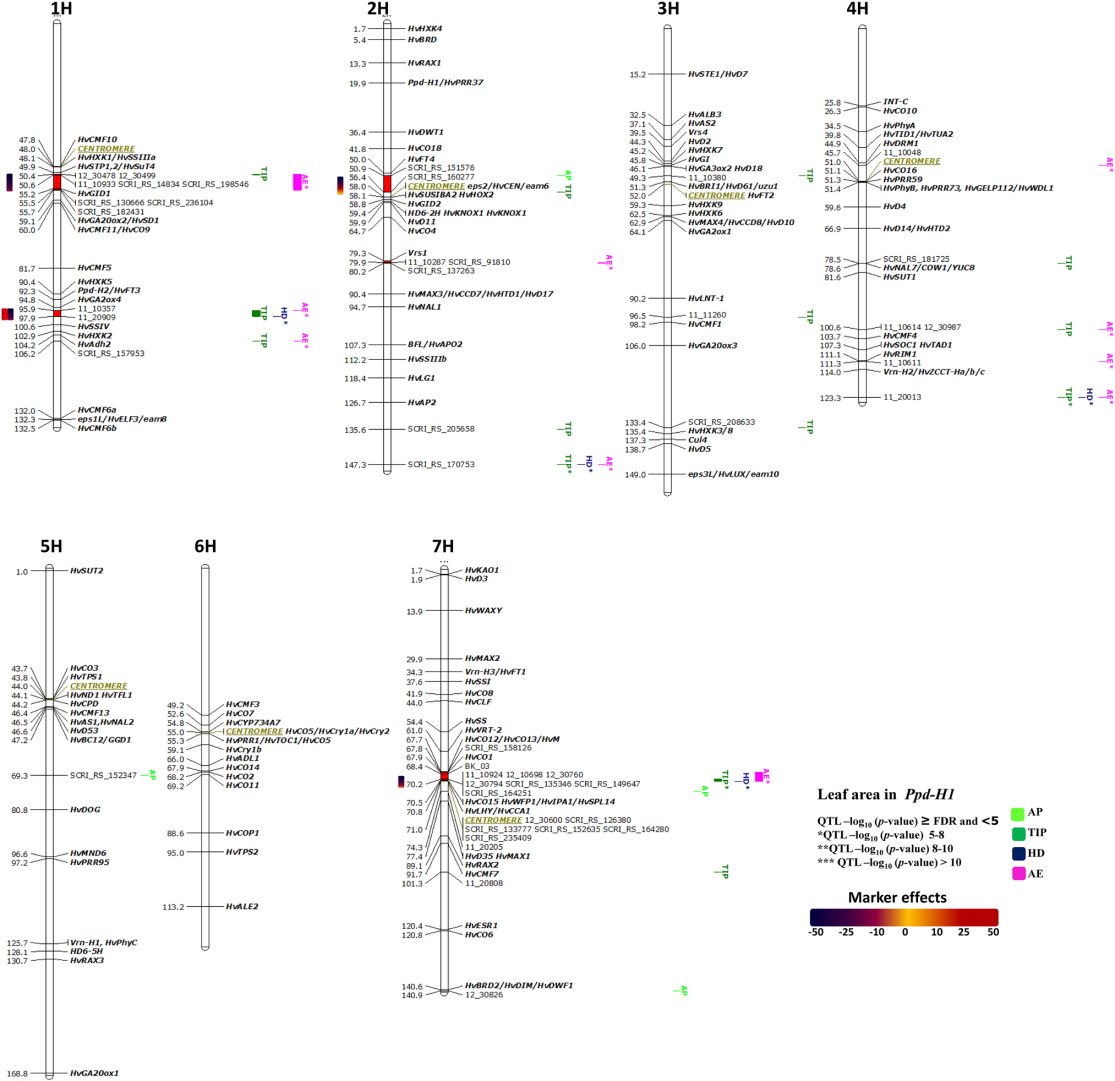
Genetically anchored position of highly associated QTL for leaf blade area per the main culm at all barley developmental stages in the photoperiod sensitive (*Ppd*-*H1*) group using 9K SNP markers. Bold and italicized gene names indicate genetically anchored positions of known heading time and plant stature genes in the Barke × Morex RILs. Associated chromosomal regions are highlighted with different colors according to developmental stages. Red chromosomal areas indicate the range of significantly associated QTL (within confidence interval ± 5 cM) which are exceeding FDR level of each developmental stage (*n* = 92 for *Ppd*-*H1* group)

The association analysis in 92 photoperiod sensitive (*Ppd*-*H1*) accessions detected four significantly associated chromosomal regions (Figure S6), of which two are without known candidate colocation on 1H, 95.9–97.9 cM and 2H, 50.9–56.4 cM (Fig. 4). These two group-specific QTL show a strong effect on LA variation at different pre-anthesis stages (Fig. 4). The associated markers of the first QTL region (1H) showed contrasting effects of markers within the QTL, indicating that there is allelic variation among the accessions of this group. The second QTL appeared at AP and TIP developmental stages and confirmed the importance of pre-anthesis stages in natural variation of LA. The remaining QTL regions very precisely co-localized with known plant stature and/or heading time genes, such as 1H, 50.4–55.7 cM, which is physically close to *TREHALOSE*-*6*-*PHOSPHATE SYNTHASE1* (*HvTPS1*), *SUCROSE TRANSPORTER4* (*HvSUT4*) and *GIBBERELLIN INSENSITIVE DWARF1* (*HvGID1*) genes, and include seven markers, suggesting a role in LA variation. The final association was around the centromeric region of 7H (67.7–71 cM; Fig. 4), including genes *CONSTANS 12/13 HvCO 12*/*HvCO 13*/*HvM*; *HvCO1*, *WEALTHY FARMERS PANICLE1/IDEAL PLANT ARCHITECTURE1/SQUAMOSA PROMOTER BINDING PROTEIN*-*LIKE14* (*HvWFP1/HvIPA1/HvSPL14*)*; LATE ELONGATED HYPOCOTYL*/*CIRCADIAN CLOCK ASSOCIATED1* (*HvLHY/HvCCA1*), showed the highest effects on LA variation. The markers in this region with – log_10_ > FDR showed negative effects (i.e. reduced LA by around − 3 cm^2^). In addition to these associations, we found two single-marker-trait associations, (2H, 147.3 and 4H, 123.3 cM); however, we could not find more associated markers to these regions most likely due to the limited number of accessions in this group. The region of *SIX*-*ROWED SPIKE 1* (*Vrs1*) gene (2H, 79.3–80.2 cM) was found to be associated with LA only causing variation at later developmental stages (AE) in this group (Thirulogachandar et al. 2017).

#### QTL for LA in the *ppd-H1* group

**Fig. 5:**
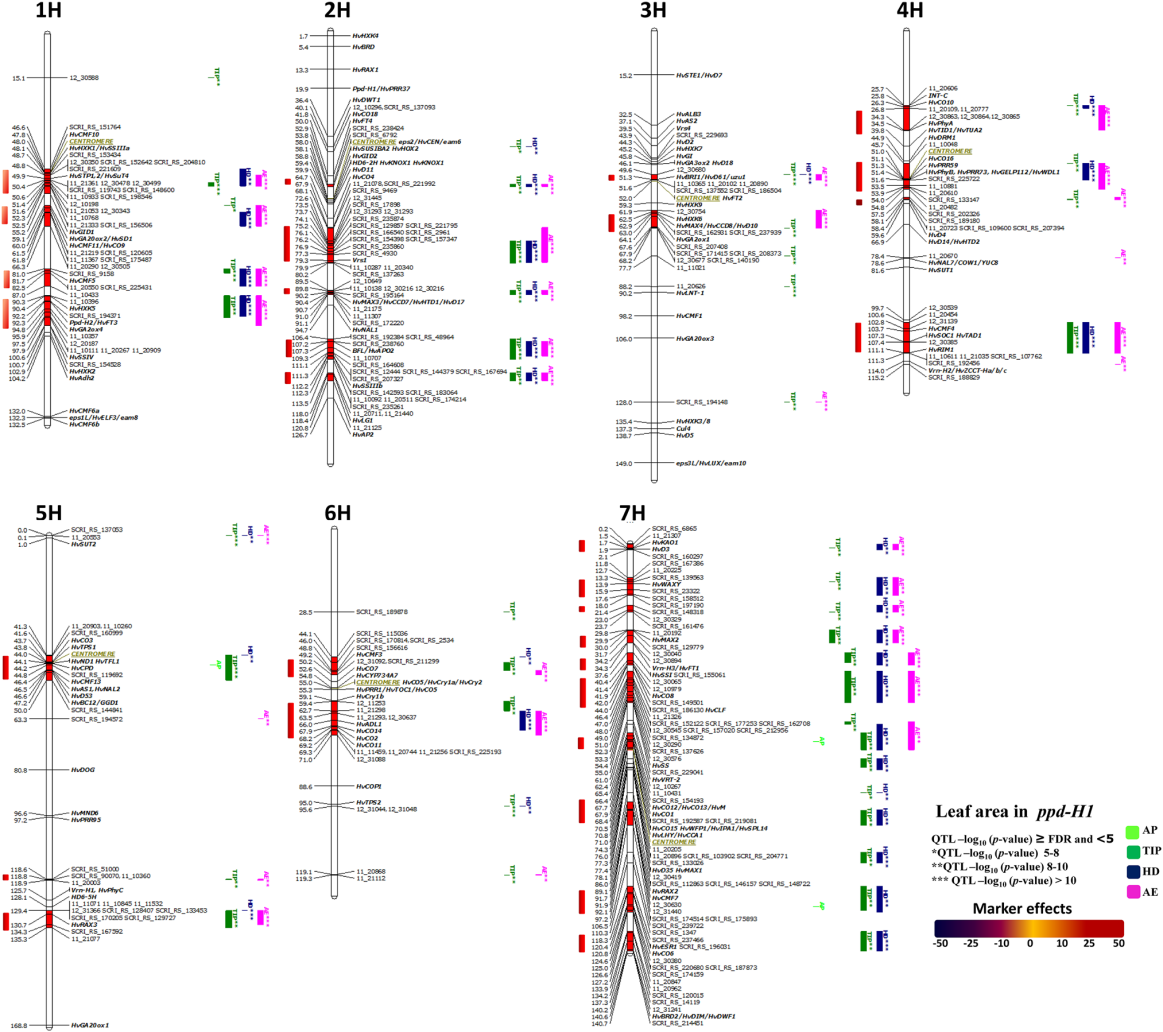
Genetically anchored position of highly associated QTL for leaf blade area per the main culm at all barley developmental stages in the reduced photoperiod sensitivity (*ppd*-*H1*) group using 9K SNP markers. Bold and italicized gene names indicate genetically anchored positions of known heading time and plant stature genes in the Barke × Morex RILs. Associated chromosomal regions are highlighted with different colors according to developmental stages. Red chromosomal areas indicate the range of significantly associated QTL (within confidence interval ± 5 cM) which are exceeding FDR level of each developmental stage (*n* = 123 for *ppd*-*H1* group)

The genetic architecture for LA was much more complex and informative in the panel of 123 accessions carrying the less functional *ppd*-*H1* allele. An important feature for this group is that the genetic variation of LA was maximized in stages after TIP (Fig. 5). Moreover, all of the highly associated markers showed positive effects on LA (i.e. increasing LA up to + 6 cm^2^; Fig. 5). Association analysis in this group showed 34 significantly associated chromosomal regions of which 29 are group-specific (Fig. 5). Seven group-specific QTL were located in the following regions: 2H, 50.0–52.9 and 118–120.8; 4H, 75.5–59.6; 5H, 0.0–0.1 and 118.6–118.9; 6H, 119.1–119.3 and 7H, 21.4–23.7 cM, demonstrating ample allelic diversity for LA in this group.

Regions exhibiting strong effects on LA in this group as shown in Fig. 5 span some potential candidate genes involved in strigolactone, brassinosteroid and gibberellin biosynthesis/metabolism, e.g. 2H 89.5–91.1, includes *HvMAX3/HvCCD7/HvHTD1/HvD17* (*MORE AXILLARY BRANCHES 3/CAROTENOID CLEAVAGE DIOXYGENASE 7/HIGH*-*TILLERING DWARF 1/DWARF 17)*; 3H 49.6–51.6 and 61.9–68.2, *HvBRI1/UZU1/HvD61* (*BRASSINOSTEROID INSENSITIVE1/SEMIBRACHYTIC*/*DWARF61*) and *HvHXK6*, *HvMAX4/CHvCD8/HvD10*, *HvGA2ox1* (*MORE AXILLARY BRANCHES 4/CAROTENOID CLEAVAGE DIOXYGENASE 8/DWARF10*, *GIBBERELLIN 20 OXIDASE 1*), respectively; 5H 129.4–135.3, *HvRAX3* (*REGULATOR OF AXILLARY MERISTEMS 3*); 7H 0.2–2.1, *HvKAO1*, *HvD3* (*ENT*-*KAURENOIC ACID HYDROXYLASE 1*, *DWARF 3*), 11.8–18.0, *WAXY*, 44.0–55.0 *HvCLF* (*CURLY LEAF*) and *HvSS1* (*SUCROSE SYNTHASE 1*), 133.9–140.7, *HvBRD2/HvDIM/HvDWF1* (*BRASSINOSTEROID DEFICIENT DWARF 2/DIMINUTO/DWARF 1*). All of these regions showed highly significant associations (− log_10_ *P* value > 8**) at the developmental stages after AP (i.e. TIP, HD and AE stages; Fig. 5).

GWAS analysis in the *ppd*-*H1* group identified putative associations of barley LA variation with sugar biosynthetic/metabolism-related candidate genes. One of these associations is located on 5H (0.0–1.0 cM) showing strong effect (− log_10_ > 10; Fig. 5) of *HvSUT2* on LA after AP stage. A second QTL (located on 6H 95.0–95.6 cM) was associated with strong effects at TIP and HD developmental stages and spanned *HvTPS2* (Fig. 5). These findings suggest a potential influence of sugar biosynthetic/metabolism related genes on LA at different developmental stages.

Six significant chromosomal regions co-localized with putative heading time genes (heading time-specific associations; Fig. 5). For instance, on 1H, 81.0–87.0 cM including *HvCMF5* (*CCT MOTIF FAMILY 5*); 4H, 25.7–34.5, *HvCO10* (*CONSTANS 10*), *HvPhyA* (*PHYTOCHROM A*); 6H, 44.1–50.2 and 59.4–71 including *HvCMF3*, *HvCO14*, *HvCO2*, *HvCO11*, respectively. These findings indicate the potential role of such genes in barley LA particularly when carrying the *ppd*-*H1* allele.

The GWAS analysis within the *ppd*-*H1* group showed seven strong associated chromosomal regions (− log_10_ > 10; Fig. 5). Two of these associated regions were located on 1H, 59.1–66.3 and 90.3–100.7 cM including *HEXOKINASE 5* (*HvHXK5*), *HvGA20ox2/HvSD1* (*GIBBERELLIN 20 OXIDASE 2/SEMIDWARF1*) and *HvCMF11/HvCO9*, and *Ppd*-*H2/HvFT3* (*PHOTOPERIOD RESPONSE LOCUS 2/FLOWERING TIME LOCUS 3*) and *HvGA2ox4*. In chromosome 4H we found another association at 100.6–111.3 cM, contains *HvCMF4*, *HvSOC1* (*SUPPRESSOR OF OVEREXPRESSION OF CONSTANS 1*), *HvTAD1* (*TILLERING AND DWARF 1*), *HvRIM1* (*RICE DWARF VIRUS MULTIPLICATION 1*). While the last four associated regions were co-located at 7H, 29.8–34.3 cM includes *HvMAX2* and *Vrn*-*H3/HvFT1*; 7H, 37.6–42.0 cM includes *SOLUBLE STARCH SYNTHASE 1* (*HvSSI*) and *HvCO8;* and 7H, 89.1–97.2 cM includes *HvRAX2* and *HvCMF7*; 118.3–127.2 includes *HvESR1* (*ENHANCER OF SHOOT REGENERATION 1*) and *HvCO6*. Moreover, it is very interesting to note detection of the region close to *BFL/HvAPO2* (*Barley FLORICAULA/LEAFY/ABERRANT PANICLE ORGANIZATION 2*; 2H, 106.4–113.5 cM) and *SOLUBLE STARCH SYNTHASE IIIb* (*HvSSIIIb*; 2H, 112.1 cM) as highly associated with LA in this group.

The analysis showed that four strong associations (− log_10_ > 8; Fig. 5) were found in the centromeric region such as 1H, 46.6–48.8 cM includes *HvCMF10* and four sugar-related genes (*HvHXK1*, *HvSSIIIb*, *HvTPS1* and *HvSUT4*). The centromeric region of 4H is physically close to *HvCO16*, *HvPRR59*, *HvPhyB*, *HvPRR73*, and *HvGELP112/HvWDL1* (*GDSL ESTERASE/LIPASE PROTEIN 112*, *WILTED DWARF AND LETHAL 1*); and 5H, 41.3–50.0 cM includes *HvCO3*, *TREHALOSE*-*6*-*PHOSPHATE SYNTHASE 1 (HvTPS1)*, *HvND1/HvTFL1/CSLD* (*NARROW LEAF AND DWARF 1/TERMINAL FLOWER 1/CURLED LEAF AND DWARF 1*), *HvCPD* (*BRASSINOSTEROID C*-*23 HYDROXYLASE*), *HvCMF13*, *HvAS1* (*ASPARAGINE SYNTHASE 1*), *HvD53*, *HvBC12/GGD1* (*BRITTLE CULM12/GIBBERELLIN*-*DEFICIENT DWARF 1*). Finally, the centromeric region of 7H, 65.4–71.2 cM contains *HvCO12*/*HvCO13*/*HvM*, *HvCO15*, *HvWFP1*/*HvIPA1*/*HvSPL14*, *HvLHY*/*HvCCA1.* Due to the strong linkage between genes in the centromeric region, it is not clear which of these genes have an effect on LA. Among these associated chromosomal regions, the SNPs located at 5H 41.3–50.0 cM (Fig. 5) showed the strongest phenotypic effect on LA by increasing LA up to + 6 cm^2^ (Fig. 5) and occurred exclusively in this group (i.e. *ppd*-*H1*).

## Discussion

The analysis of LA in our worldwide spring barley collection revealed that there is ample natural variation. Here, we measured LA at different developmental stages for better understanding the underlying genetic factors of LA based on the cumulative main culm LA which is more realistic than individual LA (e.g. flag leaf). Recently, Kebrom and Mullet (2015) highlighted the importance of main shoot leaves as the major source of sugar synthesis and photosynthetic area in sorghum. Utilizing such phenotyping approach under controlled greenhouse conditions and single plants for other cereal crops is therefore important to shed light on the genetic make-up of leaf architecture.

Digel et al. (2016) found that *Ppd*-*H1* controls leaf size in winter barley through controlling duration of cell proliferation and leaf maturation whereas the SNP22 showed the highest effect on leaf size. In the current study, the population structure using two *Ppd*-*H1* alleles (i.e. M9/SNP22 G/T) was effective in uncovering LA variation in barley. The association signals showing many *Ppd*-*H1* group-specific QTL with strong effects on LA variation for instance on 1H, 95.9–97.9 cM; 2H, 50.9–56.4 and 147.3 cM; and finally 4H, 123.3 cM. These are putatively new QTL that appeared during pre-anthesis phases in this panel, demonstrating that advanced genetic analysis for these associations is needed to understand the genetic variation of LA, when *Ppd*-*H1* alleles are functional and fully photoperiod responsive. *GID1* was previously shown in rice as soluble receptor mediating GA responses with clear impact on leaf elongation (i.e. the mutant of *GID1* produces short leaf length; Ueguchi-Tanaka et al. (2005)), the detected association around *GID1* suggests that a mutated allele of *HvGID* gene might be present in this barley panel.

The accessions carrying the less active allele of *Ppd*-*H1* (i.e. *ppd*-*H1*) showed several new group-specific QTL [e.g. (2H, 50.0–52.9 cM and 118–120.8 cM); 4H, 75.5–59.6 cM; (5H, 0.0–0.1 cM and 118.6–118.9 cM); 6H, 119.1–119.3 cM and 7H, 21.4-23.7 cM)] that only appeared here. We were unable to co-locate known candidate genes in these regions, confirming that *ppd*-*H1*-carrying accessions exhibited a complex genetic architecture and studying natural genetic variation of LA in this group is worthwhile for further genetic analysis.

Interestingly, one association controlling LA variation in the *ppd*-*H1* group is localized in the region harboring the *BFL* gene that has not been reported so far in plants. *BFL* has been implicated to be involved in phase duration and tillering in barley (Alqudah et al. 2014, 2016) and rice (Rao et al. 2008). It was shown that *RFL* (rice ortholog of *BFL*) is similarly involved in gibberellin (GA20ox), carotenoid or brassinosteroid biogenesis in rice (Rao et al. 2008). The value of these hormones in leaf development elucidated via their pivotal role in controlling leaf cells proliferation in maize and Arabidopsis (Nakaya et al. 2002; Nelissen et al. 2012) that can postulate the role of *BFL* gene in barley LA.

In the present study, genomic regions associated with LA were found to span genes involved in the strigolactone biosynthetic pathway such as *HvMAX3/HvCCD7/HvHTD1/HvD17*; and *HvMAX4/CHvCD8/HvD10*. The function of these genes is well known in maize as being influential on stem, panicle and root architecture through controlling sequential carotenoid cleavage reactions (Guan et al. 2012). Further studies are needed to evaluate the potential role of these genes in LA.

Similar conclusions can be drawn for gibberellin gene families, which have been associated with LA in the *ppd*-*H1* panel; for instance GA20ox genes (*HvGA20ox1*, *HvGA20ox2/HvSD1*, *HvGA20ox4*) highlighted the importance of these genes for LA. These enzymes are involved in the biosynthesis of active gibberellin forms that in turn may control tillering in barley (Alqudah et al. 2016) and rice plant stature (Lo et al. 2008). In addition to these genes, *HvKAO1* and *HvBC12/HvGGD1* that are strongly involved in rice plant stature by regulating the GA biosynthesis pathway (Helliwell et al. 2001; Li et al. 2011) were found to be associated with LA in our panel. These observations suggest that accessions carrying the *ppd*-*H1* allele are enriched in genetic variation for GA biosynthesis genes for LA formation. Functional analysis of the proposed link between the GA and LA will maximize our knowledge in leaf development.

Strong connections between rice leaf blade characters and *OsBRD2* were found, whereas the leaf phenotype of its mutant becomes more erect, shortened and its overexpression influences plant stature components (Hong et al. 2005). In the current study, we found marker-trait associations in the vicinity of brassinosteroid genes, for instance, *HvBRD2/HvDIM/HvDWF1*, *HvCPD*, and *HvBRI1/UZU1/HvD61*, which are crucial for plant architecture including leaf length and width (Dockter et al. 2014). The leaf phenotype of *HvBRI1* mutant (*uzu1.a*) showed a leaf-unrolling and acute leaf-blade angle as brassinosteroid signaling-deficient. In rice, *Brassinosteroid Insensitive 1* (*BRI1*)-*Associated Kinase I* (*BAK1*) has been recently reported that it regulates cell number and enlargement in leaf (Khew et al. 2015). Our GWAS results suggest that there is ample effect of brassinosteroid biosynthetic/metabolism genes on the genetic variation of LA in barley accessions carrying *ppd*-*H1* that have to be validated through further genetic analysis.

Plant stature traits, e.g. bud outgrowth in sorghum, are influenced by LA (Kebrom and Mullet 2015). In Arabidopsis, sucrose prompts leaf cell proliferation and delay the transition to cell expansion via repression of multiple chloroplast-encoded genes and up-regulation of sugar-related genes suggesting that sucrose plays a critical role in the leaf growth (Van Dingenen et al. 2016). Therefore, it might be possible to suggest that sugar-related genes in the accessions carrying-*ppd*-*H1* (i.e. late heading time) play critical role that lead for further stimulation of cell proliferation subsequently larger LA. Further molecular and genetic evidence are essential to validate the proposed link.

Interestingly, several associations were close to genes carrying single CCT [CO, CO-LIKE, TIMING OF CAB1 (TOC1)] and/or b-box domains such as *HvCMF* and *HvCO* family genes. Circadian Clocks have a substantial role in plant growth and adaptation, for instance LA of Arabidopsis short-period mutant of *TOC1* (*toc1*-*1*) showed large LA (Dodd et al. 2005). Postulating a similar role of this gene in the accessions carrying-*ppd*-*H1* allele requires advanced genetic analysis to characterize and understand its role in LA variation.

It was previously shown that the centromeric region of 7H has a strong effect on phase transition (Alqudah et al. 2014) and tillering in this population (Alqudah et al. 2016). Our findings demonstrated that this region is rich in genes that are important for natural variation of different developmental traits. Contrasting effect of highly associated markers in this region had been detected (i.e. reduced LA by − 3 cm^2^ in *Ppd*-*H1* accessions, while increased LA by + 4.5 cm^2^ in *ppd*-*H1* accession), indicating that there is allelic variation of at least one gene. Moreover, the region of 7H centromere includes the *HvGELP112/HvWDL1* gene, which regulates rice plant stature (Park et al. 2010), suggesting that this gene is potentially a candidate, especially in the *ppd*-*H1* group of accessions.

## Conclusion

LA is a complex trait that is regulated by endogenous and environmental factors; however, their interactions are not well understood. The previous lack of information about the natural variation of LA in temperate cereals increased the complexity of this trait for yield improvement. Many putative candidate gene families like *CMF*-, *CO*-like genes, sugar-related genes, strigolactones, gibberellin and brassinosteroid biosynthesis genes are proposed to be involved in LA formation. This study gives an overview regarding genetic factors controlling LA that may establish the basis for further work in cereals. More advanced molecular genetic analyses are required to validate the function of the candidate associations.

### **Author contribution statement**

Conceived the project: TS. Designed and performed the experiments: AMA, TS. Analyzed the data: AMA. Network analysis: HMY, AMA. Germplasm Resource and Genotyping: AG. Wrote the paper: AMA, TS with contributions from all co-authors.

## Electronic supplementary material

Below is the link to the electronic supplementary material.

Supplementary material 1 (DOCX 766 kb)
