# Supplementary material for: Natural variation and genetic make-up of leaf blade area in spring barley
Source: Theor Appl Genet. 2018 Jan 19;131(4):873–86. doi: 10.1007/s00122-018-3053-2 (PMC5852197; doi:10.1007/s00122-018-3053-2)
Supplement: Supplementary file 1 — Supplementary material 1 (DOCX 766 kb) [file 122_2018_3053_MOESM1_ESM.docx]

Figure S1: Population structure of 215 spring barley accessions based on 6355 SNPs information (Alqudah et al. 2014). 92 accessions showing photoperiod sensitive (*Ppd-H1*) and 123 accessions with reduced photoperiod sensitivity (*ppd-H1*).


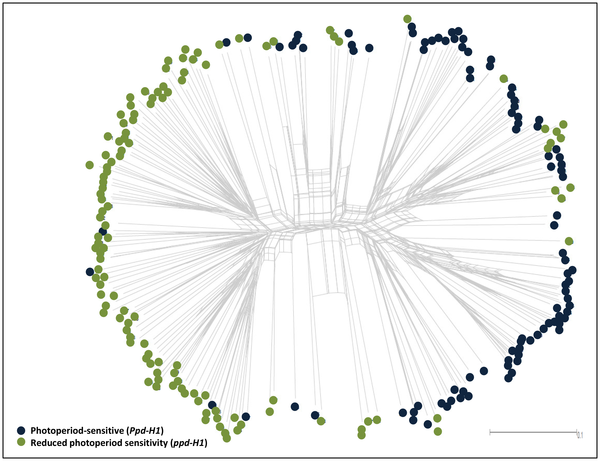


Figure S2: Correlation analysis between leaf blade area and leaf number per the main culm in both photoperiod group across all developmental stages. n = 92 accessions of photoperiod sensitive (*Ppd-H1*) and 123 for reduced photoperiod sensitivity (*ppd-H1*).


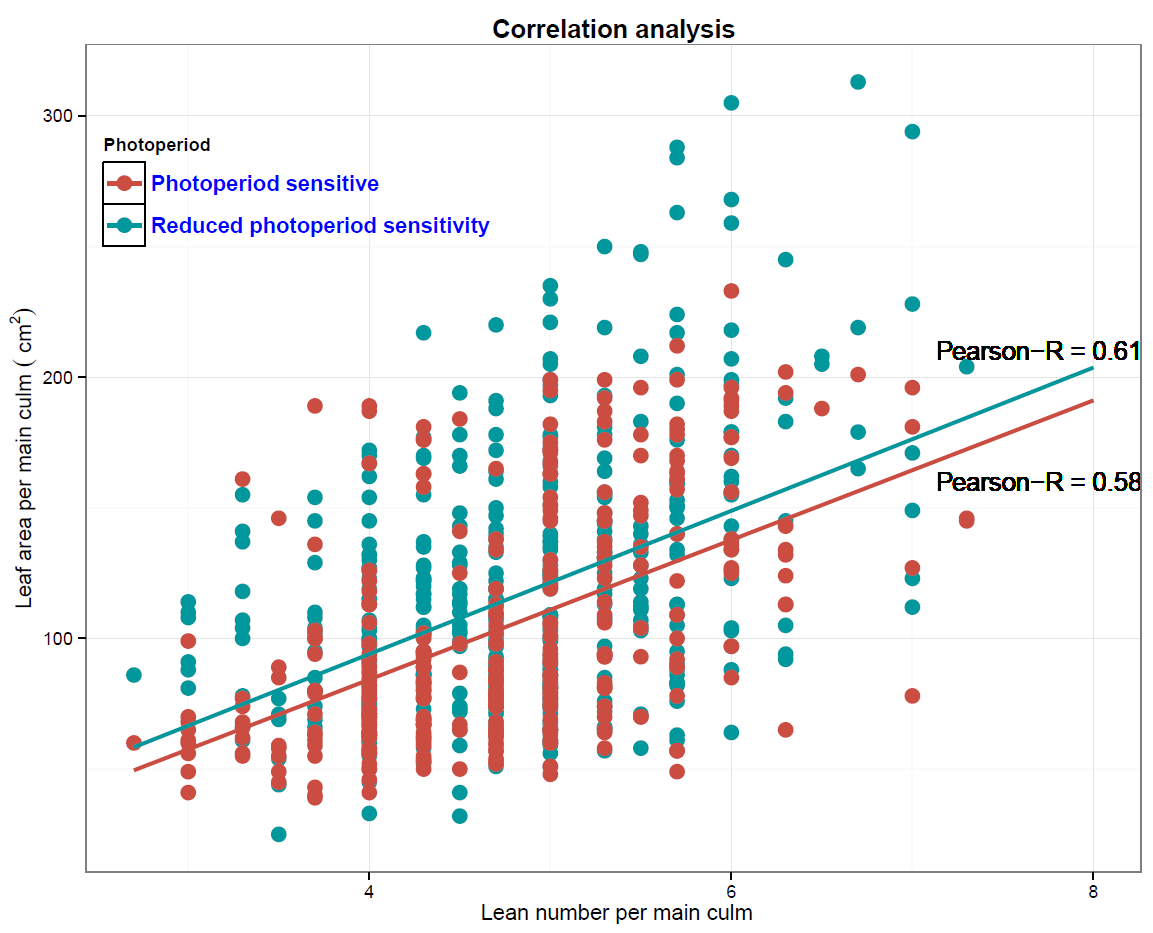


Figure S3: Leaf blade area per the main culm at different developmental stages for a) reduced photoperiod sensitivity (*ppd-H*) and b) photoperiod-sensitive (*Ppd-H1*) from different origins. Leaf area from sowing to the beginning of awn primordium, tipping, heading and anther extrusion stages. Number of *Ppd-H1*-carrying accessions for WANA = 34, EU = 13, EA = 28 and AM = 17. Number of accessions with reduced photoperiod sensitivity (*ppd-H1*) for WANA = 11, EU = 92, EA = 8 and AM = 11. Asterisk denotes leaf area significantly different at P ≤ 0.05 according to LSD between geographical regions within the same photoperiod group. The degree of significance indicated as *P, 0.05; **P, 0.01; ***P, 0.001.


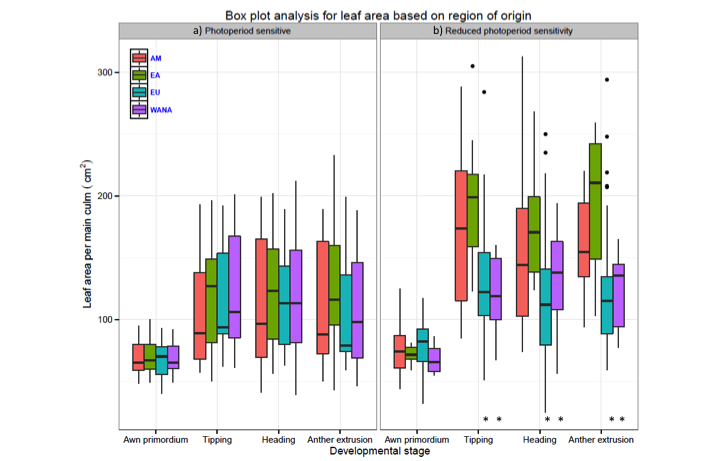


Figure S4: Leaf number per the main culm at different developmental stages of 215 spring barley accessions from different geographical origins at the same developmental stage. Asterisk denotes leaf area significantly different at P ≤ 0.05 according to LSD between geographical regions. The degree of significance indicated as *P, 0.05; **P, 0.01; ***P, 0.001. (n = 92 and 123 for *Ppd-H1* and *ppd-H1* barleys, respectively).


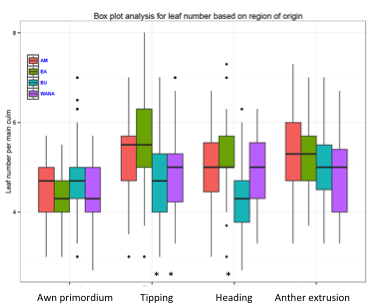


Figure S5: Principal component analysis (PCA) of 215 spring barley accessions of leaf area at tipping stage using 6355 SNPs. (n=92 spring barley accessions with photoperiod-sensitive (*Ppd-H1*) and n=123 accessions with reduced photoperiod sensitivity (*ppd-H1*)).


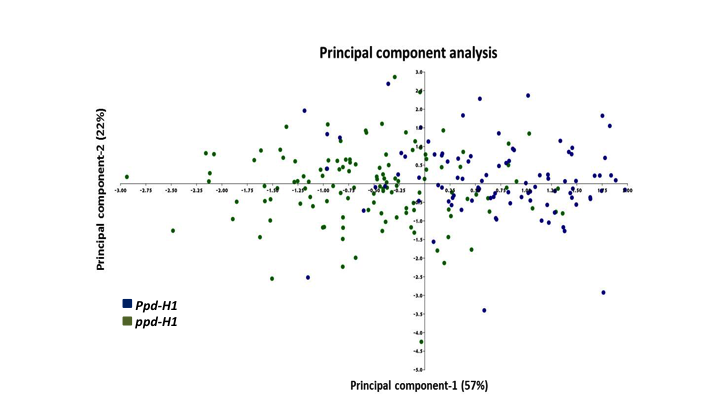


Figure S6: Manhattan plots of association findings. The figures summarize GWAS obtained leaf blade area at different stages in a) photoperiod-sensitive (*Ppd-H1*) and b) reduced photoperiod sensitivity (*ppd-H1*) barley accessions using the iSELECT 9K SNP chip. The yellow line marks the false discovery rate (FDR) threshold, and SNPs in loci exceeding this threshold are considered as significantly associated. (n=92 spring barley accessions with photoperiod-sensitive (*Ppd-H1*) and n=123 accessions with reduced photoperiod sensitivity (*ppd-H1*)).


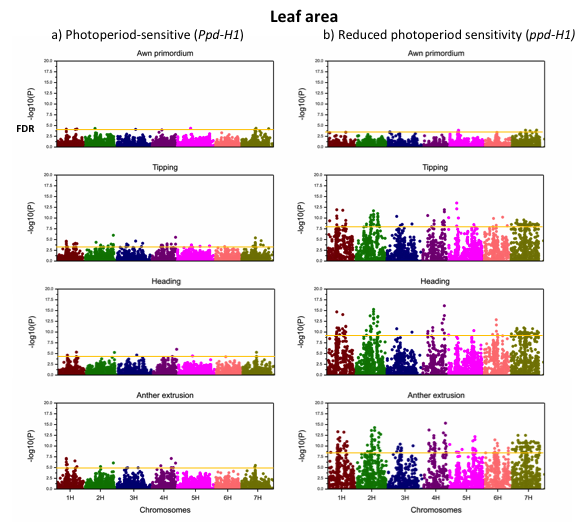


Table S1: Collection information based on origin and photoperiod-sensitivity of spring barleys accessions.

| Accession no. | Photoperiodic status | Region of origin | Country of origin | AP-Leaf area | Tip-Leaf area | Head-Leaf area | AnthExt-leaf area |
| --- | --- | --- | --- | --- | --- | --- | --- |
| BCC003 | Sensitive | WANA | AFG | 53 | 93 | 77 | 91 |
| BCC093 | Sensitive | WANA | IRQ | 49 | 61 | 39 | 55 |
| BCC118 | Sensitive | WANA | LBY | 60 | 91 | 113 | 134 |
| BCC126 | Sensitive | WANA | MAR | 64 | 106 | 109 | 130 |
| BCC129 | Sensitive | WANA | MAR | 80 | 170 | 152 | 167 |
| BCC131 | Sensitive | WANA | MAR | 62 | 109 | 122 | 125 |
| BCC1368 | Sensitive | EU | NLD | 53 | 94 | 80 | 74 |
| BCC1370 | Insensitive | EU | FRA | 86 | 151 | 25 | 39 |
| BCC1371 | Sensitive | EU | FRA | 66 | * | 143 | 79 |
| BCC1372 | Insensitive | EU | POL | 74 | 122 | 41 | * |
| BCC1373 | Insensitive | EU | GBR | 90 | 145 | 140 | 110 |
| BCC1374 | Insensitive | EU | NLD | 61 | 135 | 162 | 47 |
| BCC1376 | Insensitive | EU | DNK | 81 | * | * | * |
| BCC1377 | Insensitive | EU | FRA | 103 | 181 | 140 | 136 |
| BCC1378 | Insensitive | EU | GBR | 67 | 122 | 103 | 89 |
| BCC1379 | Insensitive | EU | CZE | 103 | 117 | 97 | 100 |
| BCC1380 | Insensitive | EU | FRA | 61 | 89 | 126 | 99 |
| BCC1381 | Insensitive | EU | GBR | 69 | 121 | 77 | * |
| BCC1382 | Insensitive | EU | GBR | 73 | 119 | * | * |
| BCC1383 | Insensitive | EU | GBR | 78 | 104 | 148 | 105 |
| BCC1384 | Insensitive | EU | DEU | 98 | 71 | 33 | * |
| BCC1385 | Insensitive | EU | POL | 75 | 117 | 118 | 60 |
| BCC1386 | Insensitive | EU | DEU | 74 | 170 | 69 | * |
| BCC1387 | Insensitive | EU | NLD | 74 | 164 | 133 | 115 |
| BCC1389 | Insensitive | EU | IRL | 105 | 159 | 119 | 112 |
| BCC1390 | Insensitive | EU | SWE | 97 | 113 | 74 | 44 |
| BCC1391 | Insensitive | EU | DEU | 104 | 99 | 54 | * |
| BCC1392 | Insensitive | EU | DNK | 83 | 97 | 143 | 77 |
| BCC1394 | Sensitive | EU | NLD | 86 | 179 | 141 | 114 |
| BCC1395 | Insensitive | EU | NLD | 71 | 131 | 138 | 88 |
| BCC1396 | Insensitive | EU | SWE | 71 | 105 | 60 | * |
| BCC1397 | Insensitive | EU | HUN | 60 | 80 | 104 | 116 |
| BCC1398 | Insensitive | EU | HUN | 74 | 110 | 100 | 105 |
| BCC1399 | Insensitive | EU | SWE | 66 | 104 | 112 | 52 |
| BCC1400 | Insensitive | EU | FRA | 117 | 178 | 129 | 93 |
| BCC1401 | Insensitive | EU | DEU | 89 | 94 | 114 | 109 |
| BCC1402 | Insensitive | EU | SWE | 108 | 110 | 57 | * |
| BCC1403 | Insensitive | EU | DEU | 65 | 155 | 169 | 61 |
| BCC1405 | Insensitive | EU | GBR | 85 | 71 | 63 | * |
| BCC1407 | Insensitive | EU | AUT | 92 | 138 | 100 | 81 |
| BCC1408 | Insensitive | EU | GBR | 92 | 107 | 127 | 92 |
| BCC1409 | Insensitive | EU | AUT | 109 | 126 | 108 | 71 |
| BCC1410 | Insensitive | EU | SWE | 81 | 79 | 86 | 62 |
| BCC1411 | Insensitive | EU | DEU | 80 | 94 | 74 | 130 |
| BCC1412 | Insensitive | EU | SWE | 90 | 81 | 77 | 97 |
| BCC1413 | Insensitive | EU | DEU | 53 | 86 | 74 | 65 |
| BCC1414 | Insensitive | EU | CZE | 109 | 109 | 102 | 75 |
| BCC1415 | Insensitive | EU | GBR | 99 | 123 | 91 | 92 |
| BCC1416 | Insensitive | EU | SWE | 97 | 113 | 90 | 93 |
| BCC1417 | Insensitive | EU | DEU | 90 | 75 | * | * |
| BCC1418 | Insensitive | EU | DNK | 92 | 190 | 142 | 67 |
| BCC1419 | Insensitive | EU | DEU | 86 | 81 | 70 | 52 |
| BCC1420 | Insensitive | EU | NLD | 77 | 67 | 73 | 66 |
| BCC1421 | Insensitive | EU | CZE | 99 | 205 | 72 | 58 |
| BCC1422 | Insensitive | EU | NLD | 66 | 128 | 86 | 86 |
| BCC1423 | Insensitive | EU | FRA | 61 | 154 | 107 | 70 |
| BCC1424 | Insensitive | EU | DEU | 82 | 119 | 102 | 95 |
| BCC1425 | Insensitive | EU | DEU | 95 | 85 | 78 | 50 |
| BCC1428 | Insensitive | EU | GBR | * | 103 | * | * |
| BCC1430 | Insensitive | EU | FRA | 108 | 151 | 125 | 55 |
| BCC1431 | Insensitive | EU | AUT | 73 | 64 | 66 | 44 |
| BCC1432 | Insensitive | EU | CZE | 71 | 160 | 113 | 85 |
| BCC1433 | Insensitive | EU | DEU | 93 | 103 | 115 | 108 |
| BCC1439 | Insensitive | EU | CZE | 82 | 100 | 99 | 113 |
| BCC1440 | Insensitive | EU | AUT | 99 | 126 | 110 | 117 |
| BCC1441 | Insensitive | EU | DEU | 84 | 105 | 66 | 103 |
| BCC1442 | Insensitive | EU | GBR | 65 | 201 | 147 | 80 |
| BCC1443 | Insensitive | EU | DEU | 100 | 123 | 102 | 99 |
| BCC1444 | Insensitive | EU | CZE | 112 | 162 | 61 | 51 |
| BCC1445 | Insensitive | EU | FRA | 90 | 153 | 155 | 102 |
| BCC1447 | Insensitive | EU | FRA | 108 | 166 | 137 | 95 |
| BCC1448 | Insensitive | EU | FIN | 83 | 170 | 177 | 158 |
| BCC1450 | Insensitive | EU | FIN | 68 | * | * | * |
| BCC1452 | Insensitive | EU | NLD | 88 | 126 | 235 | 187 |
| BCC1453 | Insensitive | EU | FIN | 83 | 197 | 178 | 228 |
| BCC1455 | Insensitive | EU | RUS | 63 | 217 | 217 | 199 |
| BCC1456 | Insensitive | EU | RUS | 82 | 130 | 133 | 75 |
| BCC1457 | Sensitive | EU | RUS | 57 | 77 | 113 | 77 |
| BCC1458 | Insensitive | EU | RUS | 88 | 115 | 87 | 43 |
| BCC1459 | Sensitive | EU | RUS | 55 | 68 | 77 | 63 |
| BCC1461 | Insensitive | EU | RUS | 71 | 137 | 170 | 135 |
| BCC1463 | Insensitive | EU | RUS | 63 | 179 | 112 | 104 |
| BCC1465 | Insensitive | EU | UKR | 97 | 115 | 120 | 126 |
| BCC1466 | Insensitive | EU | UKR | 52 | 91 | 123 | 92 |
| BCC1467 | Insensitive | EU | BLR | 62 | * | * | * |
| BCC1468 | Insensitive | WANA | KAZ | 69 | 145 | 166 | 125 |
| BCC1469 | Insensitive | WANA | KAZ | 86 | * | * | * |
| BCC1470 | Sensitive | WANA | UZB | 40 | 62 | 63 | 59 |
| BCC1471 | Insensitive | EU | ARM | 51 | 76 | 83 | 91 |
| BCC1472 | Insensitive | EU | LTU | 58 | 127 | 158 | 129 |
| BCC1474 | Sensitive | EU | UKR | 61 | 145 | 205 | 249 |
| BCC1476 | Sensitive | WANA | UZB | 54 | 93 | 64 | 60 |
| BCC1479 | Insensitive | EU | RUS | 86 | 164 | 207 | 188 |
| BCC1480 | Insensitive | EU | RUS | 63 | 155 | 137 | 150 |
| BCC1481 | Insensitive | EU | RUS | 97 | 132 | 88 | 107 |
| BCC1482 | Insensitive | EU | RUS | 58 | 104 | 133 | 85 |
| BCC1483 | Insensitive | EU | RUS | 57 | 123 | 169 | 136 |
| BCC1484 | Insensitive | EU | RUS | 83 | 135 | 188 | 163 |
| BCC1485 | Sensitive | EU | RUS | 60 | 89 | 113 | 146 |
| BCC1487 | Insensitive | EU | RUS | 92 | 114 | 167 | * |
| BCC1488 | Insensitive | EU | RUS | 95 | 284 | 250 | 274 |
| BCC149 | Sensitive | WANA | MAR | 77 | 201 | 196 | 170 |
| BCC1490 | Insensitive | EU | RUS | 32 | 63 | 61 | 62 |
| BCC1493 | Insensitive | EU | UKR | 66 | 199 | 178 | 172 |
| BCC1494 | Sensitive | WANA | KAZ | 65 | 182 | 206 | 249 |
| BCC1497 | Sensitive | WANA | KGZ | 74 | 98 | 101 | 122 |
| BCC1498 | Sensitive | WANA | UZB | 61 | 62 | 64 | 68 |
| BCC1500 | Sensitive | WANA | TJK | 65 | 91 | 172 | 163 |
| BCC1503 | Sensitive | WANA | TKM | 76 | 161 | 163 | * |
| BCC1504 | Insensitive | EU | RUS | 65 | 107 | 145 | 152 |
| BCC1505 | Sensitive | EU | UKR | 86 | 151 | 138 | 136 |
| BCC1506 | Insensitive | EU | UKR | 45 | 132 | 108 | 63 |
| BCC1524 | Insensitive | EU | DEU | 56 | 119 | 140 | 108 |
| BCC1529 | Insensitive | EU | AUT | 61 | * | * | * |
| BCC1541 | Sensitive | EU | YUG | 70 | 92 | 102 | 94 |
| BCC1561 | Sensitive | EU | BGR | 71 | 209 | 217 | 236 |
| BCC1565 | Sensitive | EU | ALB | 65 | 126 | 113 | 106 |
| BCC1566 | Insensitive | EU | GRC | 65 | 172 | 218 | * |
| BCC1589 | Insensitive | EU | ITA | 73 | 51 | 76 | * |
| BCC161 | Sensitive | WANA | MAR | 89 | 84 | 136 | 94 |
| BCC167 | Sensitive | WANA | OMN | 81 | 94 | 79 | 65 |
| BCC173 | Sensitive | WANA | PAK | 64 | 213 | 224 | 216 |
| BCC182 | Sensitive | WANA | PAK | 89 | 168 | 94 | 84 |
| BCC190 | Sensitive | WANA | SYR | 58 | 128 | 119 | 77 |
| BCC192 | Sensitive | WANA | SYR | 89 | 177 | 229 | * |
| BCC195 | Sensitive | WANA | SYR | 69 | 74 | 121 | 64 |
| BCC197 | Sensitive | WANA | SYR | 55 | 184 | 141 | 70 |
| BCC218 | Sensitive | WANA | TJK | 92 | 165 | 212 | 188 |
| BCC219 | Sensitive | WANA | TJK | 103 | 209 | 178 | 98 |
| BCC421 | Sensitive | EA | CHN | 49 | 105 | 135 | 102 |
| BCC423 | Sensitive | EA | CHN | 100 | * | * | * |
| BCC427 | Sensitive | EA | CHN | 52 | 50 | 56 | 43 |
| BCC432 | Insensitive | EA | CHN | 69 | 123 | 124 | * |
| BCC434 | Sensitive | EA | CHN | 83 | 145 | 103 | 84 |
| BCC436 | Insensitive | EA | CHN | 68 | 208 | 133 | 83 |
| BCC438 | Sensitive | EA | CHN | 67 | 78 | 84 | 100 |
| BCC439 | Sensitive | EA | CHN | 67 | 205 | 177 | 175 |
| BCC445 | Insensitive | EA | CHN | 68 | 205 | 224 | 239 |
| BCC446 | Insensitive | EA | CHN | 74 | 193 | 191 | 173 |
| BCC447 | Insensitive | EA | CHN | 77 | 305 | 268 | 227 |
| BCC502 | Sensitive | EA | CHN | 58 | 56 | 101 | 66 |
| BCC524 | Sensitive | EA | IND | 54 | 113 | 124 | 104 |
| BCC526 | Sensitive | EA | IND | 79 | 132 | 123 | 119 |
| BCC532 | Sensitive | EA | IND | 70 | 85 | 78 | 81 |
| BCC533 | Sensitive | EA | IND | 64 | 60 | 74 | 79 |
| BCC535 | Sensitive | EA | IND | 52 | 80 | 83 | 118 |
| BCC538 | Sensitive | EA | IND | 59 | 94 | 75 | 95 |
| BCC551 | Sensitive | EA | IND | 103 | 99 | 207 | * |
| BCC577 | Sensitive | EA | IND | * | 182 | 164 | 149 |
| BCC579 | Sensitive | EA | IND | 85 | 149 | 120 | 126 |
| BCC581 | Sensitive | EA | IND | 98 | 163 | 146 | 192 |
| BCC625 | Sensitive | EA | JPN | 63 | 146 | 110 | 109 |
| BCC642 | Sensitive | EA | JPN | 93 | 81 | 100 | 97 |
| BCC666 | Sensitive | EA | KOR | 66 | 127 | 123 | 140 |
| BCC667 | Sensitive | EA | KOR | 62 | 128 | 157 | 114 |
| BCC675 | Insensitive | EA | KOR | 59 | 160 | 168 | * |
| BCC695 | Sensitive | EA | KOR | 68 | 187 | 199 | 234 |
| BCC718 | Insensitive | EA | KOR | 79 | 245 | 173 | 208 |
| BCC719 | Sensitive | EA | KOR | 60 | 183 | 195 | 216 |
| BCC732 | Sensitive | EA | NPL | * | 181 | 262 | 233 |
| BCC759 | Sensitive | EA | NPL | 80 | 213 | 202 | 161 |
| BCC761 | Sensitive | EA | NPL | 115 | 234 | 242 | 163 |
| BCC766 | Sensitive | EA | NPL | 85 | 309 | 254 | 191 |
| BCC768 | Sensitive | EA | NPL | 103 | 196 | 196 | 156 |
| BCC776 | Insensitive | EA | NPL | 81 | 156 | 140 | 114 |
| BCC801 | Insensitive | AM | CAN | 72 | 119 | 103 | 74 |
| BCC806 | Sensitive | AM | MEX | 59 | * | * | * |
| BCC807 | Sensitive | AM | URY | 95 | 224 | 194 | 169 |
| BCC812 | Sensitive | AM | MEX | 48 | 58 | 68 | 50 |
| BCC814 | Sensitive | AM | USA | 69 | 147 | 134 | 88 |
| BCC817 | Sensitive | AM | USA | 49 | 57 | 50 | 70 |
| BCC818 | Sensitive | AM | USA | 103 | 205 | 240 | 187 |
| BCC844 | Sensitive | AM | COL | 70 | 134 | 171 | 180 |
| BCC846 | Sensitive | AM | USA | 93 | 138 | 131 | 145 |
| BCC847 | Sensitive | AM | USA | 59 | 96 | 88 | 88 |
| BCC852 | Insensitive | AM | CAN | 125 | 288 | 313 | 159 |
| BCC857 | Insensitive | AM | MEX | 63 | 85 | 102 | 128 |
| BCC860 | Sensitive | AM | URY | 65 | 68 | 70 | 56 |
| BCC861 | Sensitive | AM | URY | 50 | 57 | 41 | 51 |
| BCC868 | Insensitive | AM | MEX | 44 | 176 | 150 | 141 |
| BCC869 | Insensitive | AM | MEX | 60 | 104 | 107 | 114 |
| BCC875 | Insensitive | AM | USA | 60 | 221 | 197 | 179 |
| BCC881 | Insensitive | AM | CAN | * | 263 | 230 | 200 |
| BCC888 | Insensitive | AM | CAN | 83 | 219 | 183 | 184 |
| BCC892 | Sensitive | AM | BOL | 64 | * | 41 | * |
| BCC899 | Sensitive | AM | USA | 76 | 114 | 74 | * |
| BCC900 | Insensitive | AM | CHL | 88 | * | 91 | 103 |
| BCC907 | Sensitive | AM | USA | 55 | 75 | 105 | 86 |
| BCC913 | Sensitive | AM | USA | 62 | 82 | 84 | 79 |
| BCC921 | Sensitive | AM | COL | 79 | 89 | 87 | * |
| BCC927 | Sensitive | AM | PER | 87 | 193 | 163 | 189 |
| BCC929 | Insensitive | AM | CAN | 88 | 171 | 144 | 116 |
| BCC942 | Sensitive | AM | USA | 80 | 208 | 199 | 236 |
| HOR11370 | Insensitive | WANA | ISR | 57 | 141 | 130 | 123 |
| HOR11371 | Insensitive | WANA | ISR | 63 | 119 | 138 | 125 |
| HOR11372 | Insensitive | WANA | ISR | 81 | 154 | 177 | 123 |
| HOR11373 | Insensitive | WANA | ISR | 65 | 99 | 160 | * |
| HOR11374 | Insensitive | WANA | ISR | 78 | 160 | 131 | 145 |
| HOR11403 | Sensitive | EA | IND | 82 | 127 | 81 | 67 |
| HOR12830 | Sensitive | WANA | SYR | 80 | 209 | 203 | 215 |
| HOR1391 | Sensitive | EU | ROM | 93 | 192 | 189 | 176 |
| HOR1804 | Sensitive | WANA | AFG | 103 | 176 | 158 | 125 |
| HOR1842 | Sensitive | WANA | AFG | 65 | 253 | 181 | 154 |
| HOR1962 | Sensitive | na | na | 106 | 231 | 268 | 199 |
| HOR2800 | Sensitive | WANA | IRN | 71 | 78 | 65 | 69 |
| HOR2828 | Insensitive | WANA | IRN | 55 | 107 | 86 | 69 |
| HOR2829 | Insensitive | WANA | IRN | 58 | 67 | 56 | 65 |
| HOR2835 | Insensitive | WANA | IRN | 76 | 154 | 194 | 108 |
| HOR4727 | Sensitive | WANA | TUR | 92 | 187 | 189 | 167 |
| HOR7985 | Sensitive | WANA | TUR | 57 | 71 | 100 | 77 |
| HOR8006 | Sensitive | WANA | TUR | 67 | 64 | 45 | 46 |
| HOR8050 | Insensitive | WANA | TUR | 66 | 101 | 76 | 89 |
| HOR8113 | Sensitive | WANA | TUR | 64 | 78 | 83 | 89 |
| HOR8160 | Insensitive | WANA | TUR | 56 | 82 | 139 | 57 |
| HOR930 | Sensitive | WANA | TUR | 73 | 106 | 156 | 148 |

Table S2: GenBank accession number for known leaf development, heading time and plant stature candidate genes with their POPSEQ genetic position.

| **Chr.** | **Gene** | **GenBank accession number** | **Reference** | **cM (POP SEQ)** | **Contig identifier** |
| --- | --- | --- | --- | --- | --- |
|  |  |  |  |  |  |
| 1H | *HvCMF10* | [JQ791225](http://webblast.ipk-gatersleben.de/barley/blastresult.php?jobid=140120626639&opt=none) | ([Cockram et al., 2012](#_ENREF_5)) | 47.82 | morex_contig_53826 CAJW010053826 |
| 1H | *HEXOKINASE 1 (HvHXK1),*  *SOLUBLE STARCH SYNTHASE (HvSSIIIa)* | [HM037127.1](http://www.ncbi.nlm.nih.gov/nuccore/327555162?report=fasta)  [FN179377.1](http://www.ncbi.nlm.nih.gov/nuccore/FN179377.1) | ([Cho et al., 2006](#_ENREF_4)),  ([Radchuk et al., 2009](#_ENREF_31)) | 48.08 | morex_contig_42192 CAJW010042192,  morex_contig_41334 CAJW010041334 |
| 1H | *HEXOSE*  *TRANSPORTATION1, 2/SUGAR TRANSPORTER (HvSTP1,2/HvSuT4)* | [AJ534445.1](http://www.ncbi.nlm.nih.gov/nuccore/26986185?report=fasta)  [AJ534446](http://www.ncbi.nlm.nih.gov/nuccore/26986187?report=fasta) | ([Weschke et al., 2003](#_ENREF_36)) | 49.85 | morex_contig_38718 CAJW010038718 |
| 1H | *GIBBERELLIN INSENSITIVE DWARF1 (HvGID1)* | [AK074026](http://getentry.ddbj.nig.ac.jp/getentry/ddbj/AK074026?filetype=html) | <http://rice.plantbiology.msu.edu/cgi-bin/ORF_infopage.cgi?orf=LOC_Os05g33730.1> | 55.52 | morex_contig_137029 CAJW010137029 |
| 1H | *GIBBERELLIN 20 OXIDASE 2/ SEMIDWARF 1 (HvGA20ox2/HvSD1)* | [CT834906](http://getentry.ddbj.nig.ac.jp/getentry/ddbj/CT834906?filetype=html) | ([Yang et al., 2009](#_ENREF_39)) ([Sakamoto et al., 2004](#_ENREF_33)) | 59.13 | morex_contig_244138 CAJW010244138 |
| 1H | *HvCO9/HvCMF11* | [AY082965](http://webblast.ipk-gatersleben.de/barley/blastresult.php?jobid=140120596769&opt=none) | ([Griffiths et al., 2003](#_ENREF_13);[Comadran et al., 2012](#_ENREF_6)) | ~60 | morex_contig_67944 CAJW010067944 |
| 1H | *HvCMF5* | [JQ791219](http://webblast.ipk-gatersleben.de/barley/blastresult.php?jobid=140120646281&opt=none) | ([Cockram et al., 2012](#_ENREF_5)) | 81.72 | morex_contig_79857 CAJW010079857 |
| 1H | *HEXOKINASE 5 (HvHXK5)* | [HM037131.1](http://www.ncbi.nlm.nih.gov/nuccore/327555170?report=fasta) | ([Mangelsen et al., 2011](#_ENREF_24)) | 90.43 | morex_contig_38242 CAJW010038242 |
| 1H | *Ppd-H2/HvFT3* | [HM133570.1](http://webblast.ipk-gatersleben.de/barley/blastresult.php?jobid=140120721998&opt=none) | ([Casao et al., 2011](#_ENREF_3)) | 92.35 | morex_contig_2551337 CAJW012551337 |
| 1H | *GIBBERELLIN 20 OXIDASE 4 (HvGA2ox4)* | [AY551432.1](http://www.ncbi.nlm.nih.gov/nuccore/AY551432.1) | ([Spielmeyer et al., 2004](#_ENREF_34)) | 94.75 | morex_contig_1566970 CAJW011566970 |
| 1H | *SOLUBLE STARCH SYNTHASE (HvSSIV)* | [FN179379.1](http://www.ncbi.nlm.nih.gov/nuccore/229610860?report=fasta) | ([Radchuk et al., 2009](#_ENREF_31)) | 100.56 | morex_contig_48282 CAJW010048282 |
| 1H | *HEXOKINASE 2 (HvHXK2)* | [HM037128.1](http://www.ncbi.nlm.nih.gov/nuccore/327555164?report=fasta) | ([Mangelsen et al., 2011](#_ENREF_24)) | 102.94 | morex_contig_1560054 CAJW011560054 |
| 2H | *SEMI-ROLLED LEAF1(HvSRL1)* | [AK101907](http://rice.plantbiology.msu.edu/cgi-bin/ORF_infopage.cgi?orf=LOC_Os07g01240.1) | ([Xiang et al., 2012](#_ENREF_38)) | 58.05 | morex_contig_1562222 CAJW01156222 |
| 2H | *SIX-ROWED SPIKE 1 (Vrs1)* | [AB259782.1](http://www.ncbi.nlm.nih.gov/nuccore/AB259782.1) | ([Komatsuda et al., 2007](#_ENREF_21)) | 79.30 | morex_contig_135757 CAJW010135757 |
| 2H | *MORE AXILLARY BRANCHES 3/ CAROTENOID CLEAVAGE DIOXYGENASE 7/ HIGH-TILLERING DWARF 1/ DWARF 17 (HvMAX3/HvCCD7/HvHTD1/HvD17)* | [FJ957945.1](http://www.ncbi.nlm.nih.gov/nuccore/237908816) | ([Booker et al., 2004](#_ENREF_2))  <http://rice.plantbiology.msu.edu/cgi-bin/ORF_infopage.cgi?orf=LOC_Os04g46470.1> | 90.43 | morex_contig_40001 CAJW010040001 |
| 2H | *NARROW LEAF*  *1 (HvNAL1)* | [EU093963.1](http://www.ncbi.nlm.nih.gov/nuccore/EU093963.1) | ([Qi et al., 2008](#_ENREF_30)) | 94.68 | morex_contig_52862 CAJW010052862 |
| 2H | *BARLEY FLORICAULA LEAFY/ ABERRANT PANICLE ORGANIZATION2 (BFL/HvAPO2)* | [AB005620.1](http://webblast.ipk-gatersleben.de/barley/blastresult.php?jobid=140120769868&opt=none) | ([Kyozuka et al., 1998](#_ENREF_22)) | 107.36 | morex_contig_1567741 CAJW011567741 |
| 2H | *SOLUBLE STARCH SYNTHASE (HvSSIIIb)* | [FN179378.1](http://www.ncbi.nlm.nih.gov/nuccore/229610858?report=fasta) | ([Radchuk et al., 2009](#_ENREF_31)) | 112.18 | morex_contig_39371 CAJW010039371 |
| 2H | *LIGULELESS 1 (HvLG1)* | [AK068104](http://rice.plantbiology.msu.edu/cgi-bin/ORF_infopage.cgi?orf=LOC_Os04g56170.1) | <http://rice.plantbiology.msu.edu/cgi-bin/ORF_infopage.cgi?orf=LOC_Os04g56170.1> | 118.35 | morex_contig_41806 CAJW010041806 |
| 3H | *BRASSINOSTEROID INSENSITIVE 1*  */*SEMIBRACHYTIC/  *Dwarf61* (*HvBRI1/ uzu1 HvD61/)* | [AB109215.1](http://www.ncbi.nlm.nih.gov/nuccore/AB109215.1) | ([Saisho et al., 2004](#_ENREF_32)) | 51.34 | morex_contig_58772 CAJW010058772 |
| 3H | *ROOT ARCHITECTURE ASSOCIATED 1 (HvRAA1)* | [AY659938.1](http://www.ncbi.nlm.nih.gov/nuccore/AY659938) | ([Ge et al., 2004](#_ENREF_12)) | 51.55 | morex_contig_54700 CAJW010054700 |
| 3H | *HEXOKINASE* 6 (Hv*HXK6*) | [HM037132.1](http://www.ncbi.nlm.nih.gov/nuccore/327555172?report=fasta) | ([Mangelsen et al., 2011](#_ENREF_24)) | 62.53 | morex_contig_38880 CAJW010038880 |
| 3H | *MORE AXILLARY BRANCHES 4/ CAROTENOID CLEAVAGE DIOXYGENASE 8 /DWARF 10 (HvMAX4/CHvCD8/ HvD10)* | [FJ957946](http://www.ncbi.nlm.nih.gov/nuccore/FJ957946) | ([Guan et al., 2012](#_ENREF_14)) | 62.93 | morex_contig_51744 CAJW010051744 |
| 3H | *GIBBERELLIN 20 OXIDASE 1 (HvGA2ox1)* | [JQ994304.1](http://www.ncbi.nlm.nih.gov/nuccore/JQ994304.1) | ([Kebrom et al., 2013](#_ENREF_20)) | 64.16 | morex_contig_2550522 CAJW012550522 |
| 4H | *INTERMEDIUM-C (INT-C)* | [JF904738](http://www.ncbi.nlm.nih.gov/nuccore/JF904738) | ([Youssef et al., 2012](#_ENREF_40)) | 25.84 | morex_contig_5747 CAJW010005747 |
| 4H | *HvCO10* | [JQ791236](http://webblast.ipk-gatersleben.de/barley/blastresult.php?jobid=140120977259&opt=none).1 | ([Cockram et al., 2012](#_ENREF_5)) | 26.34 | morex_contig_7813 CAJW010007813 |
| 4H | *HvPhyA* | [DQ201141.1](http://webblast.ipk-gatersleben.de/barley/blastresult.php?jobid=140120997421&opt=none) | ([Szucs et al., 2006](#_ENREF_35)) | 34.56 | morex_contig_9764 CAJW010009764 |
| 4H | *GDSL ESTERASE/LIPASE PROTEIN 112, WILTED DWARF AND LETHAL 1 (HvGELP112/HvWDL1)* | [AK067429](http://getentry.ddbj.nig.ac.jp/getentry/na/AK067429/?filetype=html) | <http://rice.plantbiology.msu.edu/cgi-bin/ORF_infopage.cgi?orf=LOC_Os11g48070.1> | 51.40 | morex_contig_1569047 CAJW011569047 |
| 4H | *HvCO16* | [JQ791248.1](http://webblast.ipk-gatersleben.de/barley/blastresult.php?jobid=140121014470&opt=none) | ([Cockram et al., 2012](#_ENREF_5)) | 51.13 | morex_contig_44067 CAJW010044067 |
| 4H | *HvPRR59* | [JQ791228](http://webblast.ipk-gatersleben.de/barley/blastresult.php?jobid=140121038873&opt=none) | ([Cockram et al., 2012](#_ENREF_5)) | 51.34 | morex_contig_46739 CAJW010046739 |
| 4H | *HvphyB* | [DQ201142](http://webblast.ipk-gatersleben.de/barley/blastresult.php?jobid=140121063126&opt=none) | ([Szucs et al., 2006](#_ENREF_35)) | 51.40 | morex_contig_1557904 CAJW011557904 |
| 4H | *HvPRR73* | [JQ791230](http://webblast.ipk-gatersleben.de/barley/blastresult.php?jobid=140121052618&opt=none) | ([Cockram et al., 2012](#_ENREF_5)) | 51.40 | morex_contig_1563982 CAJW01156398 |
| 4H | *NARROW LEAF*  *7, CONSTITUTIVELY WILTED1/YUCCA-LIKE GENE 8/ FLAVIN MONOOXYGENASE*  *(HvNAL7/COW1/YUC8)* | [AK072466](http://rice.plantbiology.msu.edu/cgi-bin/ORF_infopage.cgi?orf=LOC_Os03g06654.1) | ([Fujino et al., 2008](#_ENREF_11)) | 78.61 | morex_contig_43351 CAJW010043351 |
| 4H | *HvCMF4* | [JQ791217](http://webblast.ipk-gatersleben.de/barley/blastresult.php?jobid=140121079763&opt=none) | ([Cockram et al., 2012](#_ENREF_5)) | 103.75 | morex_contig_135706 CAJW010135706 |
| 4H | *TILLERING AND DWARF 1 (HvTAD1)* | [AK070642](http://getentry.ddbj.nig.ac.jp/getentry/ddbj/AK070642?filetype=html) | <http://rice.plantbiology.msu.edu/cgi-bin/ORF_infopage.cgi?orf=LOC_Os03g03150.1> | 107.36 | morex_contig_7057 |
| 4H | *HvSOC1* | [JN673265.1](http://webblast.ipk-gatersleben.de/barley/blastresult.php?jobid=140188282299&opt=none#BL_ORD_ID:2798602) | ([Papaefthimiou et al., 2012](#_ENREF_28)) | 107.32 | barke_contig_1803142 CAJV011657847 |
| 4H | *RICE DWARF VIRUS MULTIPLICATION 1 (HvRIM1)* | [AB265821.1](http://www.ncbi.nlm.nih.gov/nuccore/AB265821) | <http://rice.plantbiology.msu.edu/cgi-bin/ORF_infopage.cgi?orf=LOC_Os03g02800.1> | 111.11 | morex_contig_65072 CAJW010065072 |
| 5H | *SUCROSE TRANSPORTER 2 (HvSUT2)* | [AJ272308.1](http://www.ncbi.nlm.nih.gov/nuccore/7024412?report=fasta) | ([Weschke et al., 2000](#_ENREF_37)) | 0.97 | morex_contig_156785 CAJW010156785 |
| 5H | *HvCO3* | [AF490473](http://webblast.ipk-gatersleben.de/barley/blastresult.php?jobid=140125439686&opt=none) | ([Griffiths et al., 2003](#_ENREF_13)) | 43.76 | morex_contig_67117 CAJW010067117 |
| 5H | *TREHALOSE-6-PHOSPHATE SYNTHASE 1 (HvTPS1)* | [HM446020.1](http://www.ncbi.nlm.nih.gov/nuccore/328671415?report=fasta) | ([Mangelsen et al., 2011](#_ENREF_24)) | 43.76 | morex_contig_1561802 CAJW011561802 |
| 5H | *BRASSINOSTEROID C-23 HYDROXYLASE (HvCPD)* | [KF360233.1](http://www.ncbi.nlm.nih.gov/nuccore/552355952) | ([Dockter et al., 2014](#_ENREF_9)) | 44.02 | morex_contig_1559549 CAJW011559549 |
| 5H | *NARROW LEAF AND DWARF 1/* *TERMINAL FLOWER1/* *CURLED LEAF AND DWARF 1 (HvND1/TFL1/CSLD)* | [AK242601.1](http://www.ncbi.nlm.nih.gov/nuccore/AK242601) | <http://rice.plantbiology.msu.edu/cgi-bin/ORF_infopage.cgi?orf=LOC_Os12g36890.1> | 44.09 | morex_contig_1576831 CAJW011576831 |
| 5H | *HISTONE DEACETYLASE 2 (HvHDA714/HDAC10)* | [AK072557.1](http://www.ncbi.nlm.nih.gov/nuccore/AK072557) | ([Hu et al., 2009](#_ENREF_16)) | 44.23 | morex_contig_45342 CAJW010045342 |
| 5H | *narrow leaf 2 (HvNAL2)* | [AM490243.1](http://www.ncbi.nlm.nih.gov/nuccore/AM490243) | ([Nardmann et al., 2007](#_ENREF_27)) | 46.45 | morex_contig_140081 CAJW010140081 |
| 5H | *HvCMF13* | [JQ791226](http://webblast.ipk-gatersleben.de/barley/blastresult.php?jobid=140125426653&opt=none).1 | ([Cockram et al., 2012](#_ENREF_5)) | 46.45 | morex_contig_1558212 CAJW011558212 |
| 5H | *ASPARAGINE SYNTHASE1 (HvAS1)* | [AF307145.1](http://www.ncbi.nlm.nih.gov/nuccore/13925885) | ([Møller et al., 2003](#_ENREF_25)) | 46.45 | morex_contig_47260 CAJW010047260 |
| 5H | *DWARF KYUSHU 3 (HvD53)* | [AK240842](http://getentry.ddbj.nig.ac.jp/getentry/ddbj/AK240842?filetype=html) | <http://rice.plantbiology.msu.edu/cgi-bin/ORF_infopage.cgi?orf=LOC_Os11g01330.1>  <http://rapdb.dna.affrc.go.jp/viewer/gene_detail/irgsp1?name=Os11t0104300-01;feature_id=2814952> | 46.59 | morex_contig_244827 CAJW010244827 |
| 5H | *BRITTLE CULM12/* *GIBBERELLIN-DEFICIENT DWARF 1 (HvBC12/GGD1)* | [AK100974](http://www.ncbi.nlm.nih.gov/nuccore/AK100974) | <http://rice.plantbiology.msu.edu/cgi-bin/ORF_infopage.cgi?orf=LOC_Os09g02650.1> | 47.22 | morex_contig_45441 CAJW010045441 |
| 5H | *BROAD LEAF1*(*BLF1*) | [KU494020.1](https://www.ncbi.nlm.nih.gov/nucleotide/1001825581?report=genbank&log$=nuclalign&blast_rank=1&RID=ADFHF1J9014) | ([Jost et al., 2016](#_ENREF_19)) | 60.83 | Morex_contig_41925 CAJW010041925 |
| 5H | *REGULATOR OF AXILLARY MERISTEMS3 (HvRAX3)* | [NP_190538.1](http://www.ncbi.nlm.nih.gov/protein/15229192) | ([Lin et al., 1999](#_ENREF_23)) | 130.69 | morex_contig_37892 |
| 6H | *HvCMF3* | [JQ791216](http://webblast.ipk-gatersleben.de/barley/blastresult.php?jobid=140125913497&opt=none) | ([Cockram et al., 2012](#_ENREF_5)) | 49.22 | morex_contig_56141 CAJW010056141 |
| 6H | *HvCry1b* | [DQ201152](http://webblast.ipk-gatersleben.de/barley/blastresult.php?jobid=140126123570&opt=none) | ([Szucs et al., 2006](#_ENREF_35)) | 59.06 | morex_contig_48345 CAJW010048345 |
| 6H | *ADAXIALIZED LEAF 1 (HvADL1)* | [AB477099.1](http://www.ncbi.nlm.nih.gov/nuccore/AB477099.1) | <http://rice.plantbiology.msu.edu/cgi-bin/ORF_infopage.cgi?orf=LOC_Os02g47970.1> | 66.07 | morex_contig_1564648 CAJW011564648 |
| 6H | *HvCO14* | [JQ791244](http://webblast.ipk-gatersleben.de/barley/blastresult.php?jobid=140126037462&opt=none) | ([Cockram et al., 2012](#_ENREF_5)) | 67.91 | morex_contig_367999 CAJW010367999 |
| 6H | *HvCO2* | [AF490469](http://webblast.ipk-gatersleben.de/barley/blastresult.php?jobid=140126056975&opt=none) | ([Griffiths et al., 2003](#_ENREF_13)) | 68.20 | morex_contig_6805 CAJW010006805 |
| 6H | *HvCO11* | [JQ791238](http://webblast.ipk-gatersleben.de/barley/blastresult.php?jobid=140126074279&opt=none) | ([Cockram et al., 2012](#_ENREF_5)) | 69.26 | morex_contig_1577721 CAJW011577721 |
| 6H | *TREHALOSE-6-PHOSPHATE SYNTHASE 2 (HvTPS2)* | [HM446021.1](http://www.ncbi.nlm.nih.gov/nuccore/HM446021.1) | ([Mangelsen et al., 2011](#_ENREF_24)) | 95.04 | morex_contig_1581754 CAJW011581754 |
| 6H | *ABNORMAL LEAF SHAPE 2 (HvALE2)* | [AK069234](http://rice.plantbiology.msu.edu/cgi-bin/ORF_infopage.cgi?orf=LOC_Os02g57080.1) | <http://rice.plantbiology.msu.edu/cgi-bin/ORF_infopage.cgi?orf=LOC_Os02g57080.1> | 113.24 | morex_contig_8203 CAJW010008203 |
| 7H | *ENT-KAURENOIC ACID HYDROXYLASE 1 (HvKAO1)* | [AF326277.1](http://www.ncbi.nlm.nih.gov/nuccore/AF318500) | ([Helliwell et al., 2001](#_ENREF_15)) | 1.71 | morex_contig_39067 CAJW010039067 |
| 7H | *DWARF 3 (HvD3), CYTOCHROME P450 88A3* | [AK069429](http://www.ncbi.nlm.nih.gov/nuccore/AK069429) | ([Consortium et al., 2003](#_ENREF_7)) | 1.91 | morex_contig_39067 CAJW010039067 |
| 7H | *WAXY* | [AF486515.1](http://webblast.ipk-gatersleben.de/barley/blastresult.php?jobid=140126586059&opt=none) | ([Patron et al., 2002](#_ENREF_29)) | 13.88 | morex_contig_49158 CAJW010049158 |
| 7H | *MORE AXILLARY BRANCHES 2 (HvMAX2)* | [AK065478](http://getentry.ddbj.nig.ac.jp/getentry/ddbj/AK065478?filetype=html) | <http://rice.plantbiology.msu.edu/cgi-bin/ORF_infopage.cgi?orf=LOC_Os06g06050.1> | 29.95 | morex_contig_134615 CAJW01013461 |
| 7H | *Vrn-H3/HvFT1* | [DQ100327](http://webblast.ipk-gatersleben.de/barley/blastresult.php?jobid=140190023192&opt=none#BL_ORD_ID:2797059) | ([Comadran et al., 2012](#_ENREF_6)) | 34.43 | morex_contig_54983 CAJW010054983 |
| 7H | *SOLUBLE STARCH SYNTHASE (HvSSI)* | [FN179374.1](http://www.ncbi.nlm.nih.gov/nuccore/229610850?report=fasta) | ([Radchuk et al., 2009](#_ENREF_31)) | 37.60 | morex_contig_58077 CAJW010058077 |
| 7H | *HvCO8* | [AY082964](http://webblast.ipk-gatersleben.de/barley/blastresult.php?jobid=140126852493&opt=none#BL_ORD_ID:3110845) | ([Griffiths et al., 2003](#_ENREF_13)) | 41.99 | morex_contig_368769 CAJW010368769 |
| 7H | *CURLY LEAF (HvCLF)* | [AK111743](http://rice.plantbiology.msu.edu/cgi-bin/ORF_infopage.cgi?orf=LOC_Os06g16390.1) | <http://rice.plantbiology.msu.edu/cgi-bin/ORF_infopage.cgi?orf=LOC_Os06g16390.1> | 44.02 | morex_contig_1564637 CAJW011564637 |
| 7H | *SUCROSE SYNTHASE(HvSS)* | [X65871.1](http://www.ncbi.nlm.nih.gov/nuccore/19105?report=fasta) | ([de la Hoz et al., 1992](#_ENREF_8)) | 54.39 | morex_contig_1561797 CAJW011561797 |
| 7H | *HvVRT-2* | [AK355370](http://webblast.ipk-gatersleben.de/barley/blastresult.php?jobid=140126820430&opt=none#BL_ORD_ID:2779415) | ([Szucs et al., 2006](#_ENREF_35)) | 61.75 | morex_contig_37339 CAJW010037339 |
| 7H | *HvCO12* | [JQ791240](http://webblast.ipk-gatersleben.de/barley/blastresult.php?jobid=140126874462&opt=none#BL_ORD_ID:2882314) | ([Cockram et al., 2012](#_ENREF_5)) | 67.77 | morex_contig_140238 CAJW010140238 |
| 7H | *HvCO13/HvM* | [JQ791242](http://webblast.ipk-gatersleben.de/barley/blastresult.php?jobid=140126885490&opt=none#BL_ORD_ID:2788072) | ([Cockram et al., 2012](#_ENREF_5)) | 67.77 | morex_contig_45996 CAJW010045996 |
| 7H | *HvCO1* | [AF490468.1](http://webblast.ipk-gatersleben.de/barley/blastresult.php?jobid=140126894698&opt=none#BL_ORD_ID:2880410) | ([Griffiths et al., 2003](#_ENREF_13)) | 67.91 | morex_contig_138334 CAJW010138334 |
| 7H | *NO APICAL MERISTEM/CUP-SHAPED COTYLEDON3 (HvNAM/CUC3)* | [DD234777.1](http://www.ncbi.nlm.nih.gov/nuccore/DD234777.1) | <http://rice.plantbiology.msu.edu/cgi-bin/ORF_infopage.cgi?orf=LOC_Os08g40030.1> | 70.12 | morex_contig_1566840 CAJW011566840 |
| 7H | *WEALTHY FARMERS PANICLE 1/*  *IDEAL PLANT ARCHITECTURE 1/SQUAMOSA PROMOTER BINDING PROTEIN-LIKE 14 (HvWFP1/HvIPA1/HvSPL14)* | [GU136674.1](http://www.ncbi.nlm.nih.gov/nuccore/GU136674.1) | ([Jiao et al., 2010](#_ENREF_18)) | 70.50 | morex_contig_45350 CAJW010045350 |
| 7H | *HvCO15* | [JQ791246](http://webblast.ipk-gatersleben.de/barley/blastresult.php?jobid=140127162385&opt=none#BL_ORD_ID:2801195) | ([Cockram et al., 2012](#_ENREF_5)) | 70.53 | morex_contig_59119 CAJW010059119 |
| 7H | *HvCCA1* | [HQ850271.1](http://webblast.ipk-gatersleben.de/barley/blastresult.php?jobid=140162611876&opt=none#BL_ORD_ID:4309371) | ([Faure et al., 2012](#_ENREF_10)) | 70.82 | morex_contig_1567295 CAJW011567295 |
| 7H | *HvLHY* | [HQ222606.1](http://webblast.ipk-gatersleben.de/barley/blastresult.php?jobid=140127006297&opt=none#BL_ORD_ID:4309371) | <http://www.uniprot.org/uniprot/E9M5R6> | 70.82 | morex_contig_1567295 CAJW011567295 |
| 7H | *HISTONE DEACETYLASE 2 (HvsHDAC2/HDA710)* | [AK068051](http://www.ncbi.nlm.nih.gov/nuccore/32978069?report=fasta) | ([Hu et al., 2009](#_ENREF_16)) | 76.41 | morex_contig_59125 CAJW010059125 |
| 7H | *DWARF 35 (HvD35), CYTOCHROME P450 701A6* | [AK066285](http://getentry.ddbj.nig.ac.jp/getentry/ddbj/AK066285?filetype=html) | ([Itoh et al., 2004](#_ENREF_17)) | 77.40 | morex_contig_1575857 CAJW011575857 |
| 7H | *REGULATOR OF AXILLARY MERISTEMS2 (HvRAX2)* | [NP_181226.1](http://www.ncbi.nlm.nih.gov/protein/15228049) | ([Lin et al., 1999](#_ENREF_23);[Müller et al., 2006](#_ENREF_26)) | 89.13 | morex_contig_274295 CAJW010274295 |
| 7H | *HvCMF7* | [JQ791222](http://webblast.ipk-gatersleben.de/barley/blastresult.php?jobid=140127179796&opt=none#BL_ORD_ID:184177) | ([Cockram et al., 2012](#_ENREF_5)) | 91.78 | morex_contig_104939 CAJW010104939 |
| 7H | *ENHANCER OF SHOOT REGENERATION (HvESR1)* | [CV063802.1](http://www.ncbi.nlm.nih.gov/nucest/CV063802.1) | ([Ali et al., 2000](#_ENREF_1)) | 120.39 | morex_contig_39720 CAJW010039720 |
| 7H | *HvCO6* | [AY082960](http://webblast.ipk-gatersleben.de/barley/blastresult.php?jobid=140127189716&opt=none#BL_ORD_ID:2749481) | ([Griffiths et al., 2003](#_ENREF_13)) | 120.82 | morex_contig_7405 CAJW010007405 |
| 7H | *BRASSINOSTEROID DEFICIENT DWARF 2/ DIMINUTO, DWARF1 (HvBRD2/HvDIM/HvDWF1)* | [AK111949](http://getentry.ddbj.nig.ac.jp/getentry/ddbj/AK111949?filetype=html) | <http://rice.plantbiology.msu.edu/cgi-bin/ORF_infopage.cgi?orf=LOC_Os10g25780.1> | 140.65 | morex_contig_37512 CAJW010037512 |

Ali, S., Holloway, B., and Taylor, W. (2000). Normalisation of cereal endosperm EST libraries for structural and functional genomic analysis. *Plant Molecular Biology Reporter* 18**,** 123-132. doi: 10.1007/BF02824020.

Booker, J., Auldridge, M., Wills, S., Mccarty, D., Klee, H., and Leyser, O. (2004). MAX3/CCD7 Is a Carotenoid Cleavage Dioxygenase Required for the Synthesis of a Novel Plant Signaling Molecule. *Current Biology* 14**,** 1232-1238. doi: <http://dx.doi.org/10.1016/j.cub.2004.06.061>.

Casao, M.C., Igartua, E., Karsai, I., Lasa, J.M., Gracia, M.P., and Casas, A.M. (2011). Expression analysis of vernalization and day-length response genes in barley (*Hordeum vulgare* L.) indicates that *VRNH2* is a repressor of *PPDH2* (*HvFT3*) under long days. *J Exp Bot* 62**,** 1939-1949. doi: 10.1093/jxb/erq382.

Cho, J.I., Ryoo, N., Ko, S., Lee, S.K., Lee, J., Jung, K.H., Lee, Y.H., Bhoo, S.H., Winderickx, J., An, G., Hahn, T.R., and Jeon, J.S. (2006). Structure, expression, and functional analysis of the hexokinase gene family in rice (*Oryza sativa* L.). *Planta* 224**,** 598-611. doi: 10.1007/s00425-006-0251-y.

Cockram, J., Thiel, T., Steuernagel, B., Stein, N., Taudien, S., Bailey, P.C., and O'sullivan, D.M. (2012). Genome dynamics explain the evolution of flowering time CCT domain gene families in the Poaceae. *PLoS One* 7**,** e45307. doi: 10.1371/journal.pone.0045307.

Comadran, J., Kilian, B., Russell, J., Ramsay, L., Stein, N., Ganal, M., Shaw, P., Bayer, M., Thomas, W., Marshall, D., Hedley, P., Tondelli, A., Pecchioni, N., Francia, E., Korzun, V., Walther, A., and Waugh, R. (2012). Natural variation in a homolog of *Antirrhinum CENTRORADIALIS* contributed to spring growth habit and environmental adaptation in cultivated barley. *Nat Genet* 44**,** 1388-1392. doi: 10.1038/ng.2447.

Consortium, T.R.F.-L.C., Team:, N.I.O.a.S.R.F.-L.C.P., Kikuchi, S., Satoh, K., Nagata, T., Kawagashira, N., Doi, K., Kishimoto, N., Yazaki, J., Ishikawa, M., Yamada, H., Ooka, H., Hotta, I., Kojima, K., Namiki, T., Ohneda, E., Yahagi, W., Suzuki, K., Li, C.J., Ohtsuki, K., Shishiki, T., Sequencing, F.O.a.O.I.S.G., Group:, A., Otomo, Y., Murakami, K., Iida, Y., Sugano, S., Fujimura, T., Suzuki, Y., Tsunoda, Y., Kurosaki, T., Kodama, T., Masuda, H., Kobayashi, M., Xie, Q., Lu, M., Narikawa, R., Sugiyama, A., Mizuno, K., Yokomizo, S., Niikura, J., Ikeda, R., Ishibiki, J., Kawamata, M., Yoshimura, A., Miura, J., Kusumegi, T., Oka, M., Ryu, R., Ueda, M., Matsubara, K., Riken:, Kawai, J., Carninci, P., Adachi, J., Aizawa, K., Arakawa, T., Fukuda, S., Hara, A., Hashidume, W., Hayatsu, N., Imotani, K., Ishii, Y., Itoh, M., Kagawa, I., Kondo, S., Konno, H., Miyazaki, A., Osato, N., Ota, Y., Saito, R., Sasaki, D., Sato, K., Shibata, K., Shinagawa, A., Shiraki, T., Yoshino, M., and Hayashizaki, Y. (2003). Collection, Mapping, and Annotation of Over 28,000 cDNA Clones from japonica Rice. *Science* 301**,** 376-379. doi: 10.1126/science.1081288.

De La Hoz, P.S., Vicente-Carbajosa, J., Mena, M., and Carbonero, P. (1992). Homologous sucrose synthase genes in barley (Hordeum vulgare) are located in chromosomes 7H (syn. 1 and 2H Evidence for a gene translocation? *FEBS Letters* 310**,** 46-50. doi: <http://dx.doi.org/10.1016/0014-5793(92)81143-A>.

Dockter, C., Gruszka, D., Braumann, I., Druka, A., Druka, I., Franckowiak, J., Gough, S.P., Janeczko, A., Kurowska, M., Lundqvist, J., Lundqvist, U., Marzec, M., Matyszczak, I., Muller, A.H., Oklestkova, J., Schulz, B., Zakhrabekova, S., and Hansson, M. (2014). Induced variations in brassinosteroid genes define barley height and sturdiness, and expand the green revolution genetic toolkit. *Plant Physiol* 166**,** 1912-1927. doi: 10.1104/pp.114.250738.

Faure, S., Turner, A.S., Gruszka, D., Christodoulou, V., Davis, S.J., Von Korff, M., and Laurie, D.A. (2012). Mutation at the circadian clock gene *EARLY MATURITY 8* adapts domesticated barley (*Hordeum vulgare*) to short growing seasons. *Proc Natl Acad Sci U S A* 109**,** 8328-8333. doi: 10.1073/pnas.1120496109.

Fujino, K., Matsuda, Y., Ozawa, K., Nishimura, T., Koshiba, T., Fraaije, M., and Sekiguchi, H. (2008). NARROW LEAF 7 controls leaf shape mediated by auxin in rice. *Molecular Genetics and Genomics* 279**,** 499-507. doi: 10.1007/s00438-008-0328-3.

Ge, L., Chen, H., Jiang, J.-F., Zhao, Y., Xu, M.-L., Xu, Y.-Y., Tan, K.-H., Xu, Z.-H., and Chong, K. (2004). Overexpression of OsRAA1 Causes Pleiotropic Phenotypes in Transgenic Rice Plants, including Altered Leaf, Flower, and Root Development and Root Response to Gravity. *Plant Physiology* 135**,** 1502-1513. doi: 10.1104/pp.104.041996.

Griffiths, S., Dunford, R.P., Coupland, G., and Laurie, D.A. (2003). The evolution of CONSTANS-like gene families in barley, rice, and Arabidopsis. *Plant Physiol* 131**,** 1855-1867. doi: 10.1104/pp.102.016188.

Guan, J.C., Koch, K.E., Suzuki, M., Wu, S., Latshaw, S., Petruff, T., Goulet, C., Klee, H.J., and Mccarty, D.R. (2012). Diverse Roles of Strigolactone Signaling in Maize Architecture and the Uncoupling of a Branching-Specific Subnetwork. *Plant Physiology* 160**,** 1303-1317. doi: 10.1104/pp.112.204503.

Helliwell, C.A., Chandler, P.M., Poole, A., Dennis, E.S., and Peacock, W.J. (2001). The CYP88A cytochrome P450, ent-kaurenoic acid oxidase, catalyzes three steps of the gibberellin biosynthesis pathway. *Proceedings of the National Academy of Sciences* 98**,** 2065-2070. doi: 10.1073/pnas.98.4.2065.

Hu, Y., Qin, F., Huang, L., Sun, Q., Li, C., Zhao, Y., and Zhou, D.-X. (2009). Rice histone deacetylase genes display specific expression patterns and developmental functions. *Biochemical and Biophysical Research Communications* 388**,** 266-271. doi: <http://dx.doi.org/10.1016/j.bbrc.2009.07.162>.

Itoh, H., Tatsumi, T., Sakamoto, T., Otomo, K., Toyomasu, T., Kitano, H., Ashikari, M., Ichihara, S., and Matsuoka, M. (2004). A Rice Semi-Dwarf Gene, Tan-Ginbozu (D35), Encodes the Gibberellin Biosynthesis Enzyme, ent-Kaurene Oxidase. *Plant Molecular Biology* 54**,** 533-547. doi: 10.1023/B:PLAN.0000038261.21060.47.

Jiao, Y., Wang, Y., Xue, D., Wang, J., Yan, M., Liu, G., Dong, G., Zeng, D., Lu, Z., Zhu, X., Qian, Q., and Li, J. (2010). Regulation of *OsSPL14* by *OsmiR156* defines ideal plant architecture in rice. *Nat Genet* 42**,** 541-544. doi: 10.1038/ng.591.

Jost, M., Hensel, G., Kappel, C., Druka, A., Sicard, A., Hohmann, U., Beier, S., Himmelbach, A., Waugh, R., Kumlehn, J., Stein, N., and Lenhard, M. (2016). The INDETERMINATE DOMAIN Protein BROAD LEAF1 Limits Barley Leaf Width by Restricting Lateral Proliferation. *Curr Biol* 26**,** 903-909. doi: 10.1016/j.cub.2016.01.047.

Kebrom, T.H., Spielmeyer, W., and Finnegan, E.J. (2013). Grasses provide new insights into regulation of shoot branching. *Trends Plant Sci* 18**,** 41-48. doi: 10.1016/j.tplants.2012.07.001.

Komatsuda, T., Pourkheirandish, M., He, C., Azhaguvel, P., Kanamori, H., Perovic, D., Stein, N., Graner, A., Wicker, T., Tagiri, A., Lundqvist, U., Fujimura, T., Matsuoka, M., Matsumoto, T., and Yano, M. (2007). Six-rowed barley originated from a mutation in a homeodomain-leucine zipper I-class homeobox gene. *Proc Natl Acad Sci U S A* 104**,** 1424-1429. doi: 10.1073/pnas.0608580104.

Kyozuka, J., Konishi, S., Nemoto, K., Izawa, T., and Shimamoto, K. (1998). Down-regulation of *RFL*, the *FLO*/*LFY* homolog of rice, accompanied with panicle branch initiation. *Proc Natl Acad Sci U S A* 95**,** 1979-1982.

Lin, X., Kaul, S., Rounsley, S., Shea, T.P., Benito, M.-I., Town, C.D., Fujii, C.Y., Mason, T., Bowman, C.L., Barnstead, M., Feldblyum, T.V., Buell, C.R., Ketchum, K.A., Lee, J., Ronning, C.M., Koo, H.L., Moffat, K.S., Cronin, L.A., Shen, M., Pai, G., Van Aken, S., Umayam, L., Tallon, L.J., Gill, J.E., Adams, M.D., Carrera, A.J., Creasy, T.H., Goodman, H.M., Somerville, C.R., Copenhaver, G.P., Preuss, D., Nierman, W.C., White, O., Eisen, J.A., Salzberg, S.L., Fraser, C.M., and Venter, J.C. (1999). Sequence and analysis of chromosome 2 of the plant Arabidopsis thaliana. *Nature* 402**,** 761-768.

Mangelsen, E., Kilian, J., Harter, K., Jansson, C., Wanke, D., and Sundberg, E. (2011). Transcriptome Analysis of High-Temperature Stress in Developing Barley Caryopses: Early Stress Responses and Effects on Storage Compound Biosynthesis. *Molecular Plant* 4**,** 97-115. doi: <http://dx.doi.org/10.1093/mp/ssq058>.

Møller, M.G., Taylor, C., Rasmussen, S.K., and Holm, P.B. (2003). Molecular cloning and characterisation of two genes encoding asparagine synthetase in barley (Hordeum vulgare L.). *Biochimica et Biophysica Acta (BBA) - Gene Structure and Expression* 1628**,** 123-132. doi: <http://dx.doi.org/10.1016/S0167-4781(03)00137-4>.

Müller, D., Schmitz, G., and Theres, K. (2006). Blind Homologous R2R3 Myb Genes Control the Pattern of Lateral Meristem Initiation in Arabidopsis. *The Plant Cell Online* 18**,** 586-597. doi: 10.1105/tpc.105.038745.

Nardmann, J., Zimmermann, R., Durantini, D., Kranz, E., and Werr, W. (2007). WOX Gene Phylogeny in Poaceae: A Comparative Approach Addressing Leaf and Embryo Development. *Molecular Biology and Evolution* 24**,** 2474-2484. doi: 10.1093/molbev/msm182.

Papaefthimiou, D., Kapazoglou, A., and Tsaftaris, A.S. (2012). Cloning and characterization of *SOC1* homologs in barley (*Hordeum vulgare*) and their expression during seed development and in response to vernalization. *Physiol Plant* 146**,** 71-85. doi: 10.1111/j.1399-3054.2012.01610.x.

Patron, N.J., Smith, A.M., Fahy, B.F., Hylton, C.M., Naldrett, M.J., Rossnagel, B.G., and Denyer, K. (2002). The altered pattern of amylose accumulation in the endosperm of low-amylose barley cultivars is attributable to a single mutant allele of granule-bound starch synthase I with a deletion in the 5'-non-coding region. *Plant Physiol* 130**,** 190-198. doi: 10.1104/pp.005454.

Qi, J., Qian, Q., Bu, Q., Li, S., Chen, Q., Sun, J., Liang, W., Zhou, Y., Chu, C., Li, X., Ren, F., Palme, K., Zhao, B., Chen, J., Chen, M., and Li, C. (2008). Mutation of the Rice Narrow leaf1 Gene, Which Encodes a Novel Protein, Affects Vein Patterning and Polar Auxin Transport. *Plant Physiology* 147**,** 1947-1959. doi: 10.1104/pp.108.118778.

Radchuk, V.V., Borisjuk, L., Sreenivasulu, N., Merx, K., Mock, H.-P., Rolletschek, H., Wobus, U., and Weschke, W. (2009). Spatiotemporal Profiling of Starch Biosynthesis and Degradation in the Developing Barley Grain. *Plant Physiology* 150**,** 190-204. doi: 10.1104/pp.108.133520.

Saisho, D., Tanno, K., Chono, M., Honda, I., Kitano, H., and Takeda, K. (2004). Spontaneous Brassinolide-insensitive barley mutants 'uzu' adapted to East Asia. *Breeding Science* 54**,** 409-416. doi: 10.1270/jsbbs.54.409.

Sakamoto, T., Miura, K., Itoh, H., Tatsumi, T., Ueguchi-Tanaka, M., Ishiyama, K., Kobayashi, M., Agrawal, G.K., Takeda, S., Abe, K., Miyao, A., Hirochika, H., Kitano, H., Ashikari, M., and Matsuoka, M. (2004). An Overview of Gibberellin Metabolism Enzyme Genes and Their Related Mutants in Rice. *Plant Physiology* 134**,** 1642-1653. doi: 10.1104/pp.103.033696.

Spielmeyer, W., Ellis, M., Robertson, M., Ali, S., Lenton, J., and Chandler, P. (2004). Isolation of gibberellin metabolic pathway genes from barley and comparative mapping in barley, wheat and rice. *Theoretical and Applied Genetics* 109**,** 847-855. doi: 10.1007/s00122-004-1689-6.

Szucs, P., Karsai, I., Von Zitzewitz, J., Meszaros, K., Cooper, L.L., Gu, Y.Q., Chen, T.H., Hayes, P.M., and Skinner, J.S. (2006). Positional relationships between photoperiod response QTL and photoreceptor and vernalization genes in barley. *Theor Appl Genet* 112**,** 1277-1285. doi: 10.1007/s00122-006-0229-y.

Weschke, W., Panitz, R., Gubatz, S., Wang, Q., Radchuk, R., Weber, H., and Wobus, U. (2003). The role of invertases and hexose transporters in controlling sugar ratios in maternal and filial tissues of barley caryopses during early development. *The Plant Journal* 33**,** 395-411. doi: 10.1046/j.1365-313X.2003.01633.x.

Weschke, W., Panitz, R., Sauer, N., Wang, Q., Neubohn, B., Weber, H., and Wobus, U. (2000). Sucrose transport into barley seeds: molecular characterization of two transporters and implications for seed development and starch accumulation. *The Plant Journal* 21**,** 455-467. doi: 10.1046/j.1365-313x.2000.00695.x.

Xiang, J.-J., Zhang, G.-H., Qian, Q., and Xue, H.-W. (2012). SEMI-ROLLED LEAF1 Encodes a Putative Glycosylphosphatidylinositol-Anchored Protein and Modulates Rice Leaf Rolling by Regulating the Formation of Bulliform Cells. *Plant Physiology* 159**,** 1488-1500. doi: 10.1104/pp.112.199968.

Yang, Y.H., Zhang, F.M., and Ge, S. (2009). Evolutionary rate patterns of the Gibberellin pathway genes. *BMC Evol Biol* 9**,** 206. doi: 10.1186/1471-2148-9-206.

Youssef, H.M., Koppolu, R., and Schnurbusch, T. (2012). Re-sequencing of vrs1 and int-c loci shows that labile barleys (Hordeum vulgare convar. labile) have a six-rowed genetic background. *Genetic Resources and Crop Evolution* 59**,** 1319-1328. doi: 10.1007/s10722-011-9759-5.

Table S3: Analysis of variance (*P* ≥ 0.05) of leaf blade area at different developmental stages between genotypes, a region of origin and photoperiod status.

| Variance component | Leaf area at stage | | | | | | | | |
| --- | --- | --- | --- | --- | --- | --- | --- | --- | --- |
|  | **AP**† | | **TIP TIP** | | **HD** | | **AE** | | |
|  | Var. | F pr. | Var. | F pr. | Var. | F pr. | Var. | F pr. |  |
| Accessions | 8.461 | <0.001 | 12.93 | < 0.001 | 2.78 | < 0.001 | 12.99 | < 0.001 |  |
| Region of origin | 5.461 | 0.001 | 1.96 | 0.0091 | 1.82 | 0.0144 | 4.691 | 0.0041 |  |
| Photoperiod status | 14.61 | < 0.001 | 7.03 | <0.001 | 0.52 | 0.0025 | 0.611 | 0.0035 |  |

The population includes 215 accessions; n= 92 accessions of photoperiod sensitive (*Ppd-H1*) and n= 123 accessions with reduced photoperiod sensitivity (*ppd-H1*). †AP: awn primordium, Alqudah and Schnurbusch (2014); TIP: tipping, Z49; HD: heading, Z55; AE: anther extrusion, Z65; Hrv: Harvesting, Zadoks et al. (1974).
